# Current Status and Associated Factors of Technophobia Toward Digital Health Technologies Among Older Patients With Stroke: Mixed Methods Study

**DOI:** 10.2196/95921

**Published:** 2026-09-25

**Authors:** Yingjie Yang, Yongye Wu, Zhuoyun Wu, Yang Wu, Zilu Zhang, Yuxia Li

**Affiliations:** 1 School of Nursing, Shanghai University of Traditional Chinese Medicine, No.1200 Cailun Road, Pudong New Area, Shanghai, 201203, China, 86 21 5132 3097

**Keywords:** older adults, stroke, technophobia, digital health technologies, mixed methods

## Abstract

**Background:**

Digital health technologies increasingly support long-term stroke management and rehabilitation, but technophobia may hinder their acceptance and sustained use among older patients with stroke. Evidence in this population remains limited.

**Objective:**

This study aimed to examine the level, associated factors, and subjective experiences of technophobia toward digital health technologies among older patients with stroke, and inform targeted interventions.

**Methods:**

We used an explanatory sequential mixed methods design. In the quantitative phase, older patients with stroke were recruited by convenience sampling from a tertiary grade A hospital in Shanghai, China, between January 15, 2024, and January 2, 2025. Participants completed a general information questionnaire and the Chinese versions of the Technophobia Scale, the eHealth Literacy Scale, the Perceived Social Support Scale, and the Stroke Self-Efficacy Questionnaire. Univariable, correlation, and multivariable linear regression analyses were performed. Qualitative participants were purposively selected from the quantitative sample using maximum variation sampling informed by quantitative findings. Semistructured interviews conducted between February 6 and April 30, 2025, were analyzed using reflexive thematic analysis. Quantitative and qualitative findings were integrated to elucidate associated factors and their contextual manifestations.

**Results:**

Among 343 quantitative participants, the median technophobia score was 25.0 (IQR 22.0‐33.0). Multivariable linear regression showed lower technophobia scores among patients with monthly per capita household income of Renminbi (Chinese yuan; RMB) 5000-6999 (RMB 1=US $0.14 as of January 29, 2026) versus less than RMB 1000 (*B*=−3.60, 95% CI −6.44 to −0.76; *P*=.01) and among occasional versus never users of digital health technologies (*B*=−3.98, 95% CI −6.94 to −1.03; *P*=.008). Higher eHealth literacy (*B*=−0.45, 95% CI −0.58 to −0.32; *P*<.001), perceived social support (*B*=−0.35, 95% CI −0.42 to −0.29; *P*<.001), and stroke self-efficacy (*B*=−0.08, 95% CI −0.13 to −0.02; *P*=.01) were also associated with lower technophobia scores. Fifteen qualitative participants were interviewed, yielding 4 themes: affordability of digital health technologies, adoption and proficiency in using digital health technologies, influence of social relationship networks, and resources for digital health technology competence. Integrated analysis indicated broad convergence and complementarity between the quantitative and qualitative findings.

**Conclusions:**

Technophobia varied among older patients with stroke and was associated with monthly per capita household income, frequency of digital health technology use, eHealth literacy, perceived social support, and stroke self-efficacy. These findings suggest that technophobia is not limited to operational difficulties but may reflect the interplay of personal resources, social support, and technology use contexts. Stroke-specific digital health interventions may need to address eHealth literacy, stroke self-efficacy, social support, and user experience to promote acceptance and use among older patients with stroke.

## Introduction

Stroke is one of the leading causes of death and disability among adults worldwide and is characterized by high incidence, high disability, high mortality, high recurrence, and substantial economic burden [1,2]. It is projected that, by 2050, the global number of stroke-related deaths will reach 9.7 million, of which 8.8 million will occur among individuals aged 60 years and older [3]. Previous studies have also shown that approximately 67% of stroke deaths occur in people aged 70 years and older [4]. Older patients with stroke are often affected by age-related decline in organ function, multimorbidity, and limited functional recovery, resulting in complex clinical conditions, prolonged rehabilitation trajectories, and substantial long-term health management needs. Disease management is regarded as an important approach to improving health outcomes in patients with stroke [5]. Accordingly, improving the efficiency of long-term poststroke health management and optimizing rehabilitation outcomes have become important priorities in the care of older patients with stroke.

With the development of information technology and mobile internet, digital health technologies have gradually been integrated into stroke management. These technologies can provide real-time, dynamic, and integrated health care services and offer favorable cost-effectiveness [6]. The World Health Organization defines digital health technologies as software- and hardware-based technologies applied in the health sector to support the maintenance or continuous improvement of health, including mHealth (mobile health) apps, intelligent health platforms, and wearable devices [7]. Existing evidence suggests that digital health technologies can improve the continuity and accessibility of health services and promote patients’ self-care behaviors and healthy lifestyles [8]. For patients with stroke who require long-term follow-up, rehabilitation training, and continuous health monitoring, digital health technologies are of considerable value. However, the widespread availability of digital health technologies does not necessarily translate into smooth acceptance and sustained use among older patients with stroke.

Because of age-related decline in cognitive, psychological, and social functioning, together with poststroke impairments in motor, language, or executive function, older patients with stroke may be more susceptible to technophobia toward digital health technologies when accessing and using these technologies [9,10]. Technophobia generally refers to irrational anxiety and fear in response to technology, manifested as emotional reactions such as fear and tension, and may further lead to technology avoidance [11]. In older patients with stroke, however, the term “irrational” should be interpreted cautiously in light of disease characteristics and the context of technology use; technology-related fear in this population may coexist with functional limitations, cognitive burden, financial concerns, insufficient support, and previous negative experiences with technology [9,10,12]. Accordingly, technophobia should not be equated with deficits in digital skills, low eHealth literacy, technology anxiety, technology resistance, low technology acceptance, or digital exclusion. In this study, the Chinese version of the Technophobia Scale (TS) was used to operationalize technophobia toward digital health technologies, primarily capturing technology-related tension, technology-related fear, and concerns about privacy and security, while treating technology avoidance as a possible behavioral manifestation rather than an equivalent concept. Previous studies have shown that technophobia may undermine older adults’ acceptance and sustained use of digital health technologies and may also impair their engagement in health-promoting behaviors and their ability to cope with health-related challenges [13-18].

Existing evidence suggests that technophobia among older adults remains a concern and is associated with sociodemographic characteristics such as age, educational level, and economic status [19-23], as well as with factors including eHealth literacy, stroke self-efficacy, and social support [22,24-28]. Qualitative studies further indicate that insufficient digital skills training, poor compatibility of digital health devices, operational difficulties, concerns about privacy breaches, and uncertainty regarding the effectiveness of health care delivered through digital health technologies may all intensify older adults’ negative experiences when using digital health technologies [29-35]. However, existing research has focused primarily on the general older population [22], older adults with chronic conditions [25], mHealth accessibility barriers [12], or quantitative correlates of technophobia in specific stroke subgroups [36]. By comparison, the subjective experiences of technophobia toward digital health technologies among older patients with stroke and how related factors are manifested in actual technology use contexts remain insufficiently understood. An explanatory sequential mixed methods design is therefore needed to integrate quantitative correlates with qualitative experiential data and thereby generate a more contextualized understanding of technophobia toward digital health technologies among older patients with stroke.

From the perspective of the problem’s attributes, technophobia toward digital health technologies among older patients with stroke is unlikely to arise solely from deficits in individual capability. Rather, it is more plausibly the product of interactions among individual characteristics, interpersonal support, and the context of technology use. The socioecological perspective emphasizes that health-related behaviors and their psychological responses are shaped by multiple influences operating together [37] and thus provides an appropriate analytic framework for understanding technophobia toward digital health technologies among older patients with stroke. Guided by this perspective, this study systematically examined the level, associated factors, and subjective experiences of technophobia toward digital health technologies among older patients with stroke at the individual level (eg, eHealth literacy and stroke self-efficacy), the interpersonal support level (eg, perceived social support), and the technology use context level (eg, monthly per capita household income and use of home-based health management devices) to inform the development of appropriate interventions to alleviate technophobia and improve the efficiency of health care delivery for older patients with stroke. The specific objectives were (1) to describe the level of technophobia toward digital health technologies among older patients with stroke using a cross-sectional questionnaire survey and to identify associated factors, and (2) to explore, through semistructured interviews, the subjective experiences of technophobia toward digital health technologies among older patients with stroke and the contextual factors they perceived as related to those experiences, thereby complementing and helping to explain the quantitative findings.

## Methods

### Study Design

**Figure 1.**
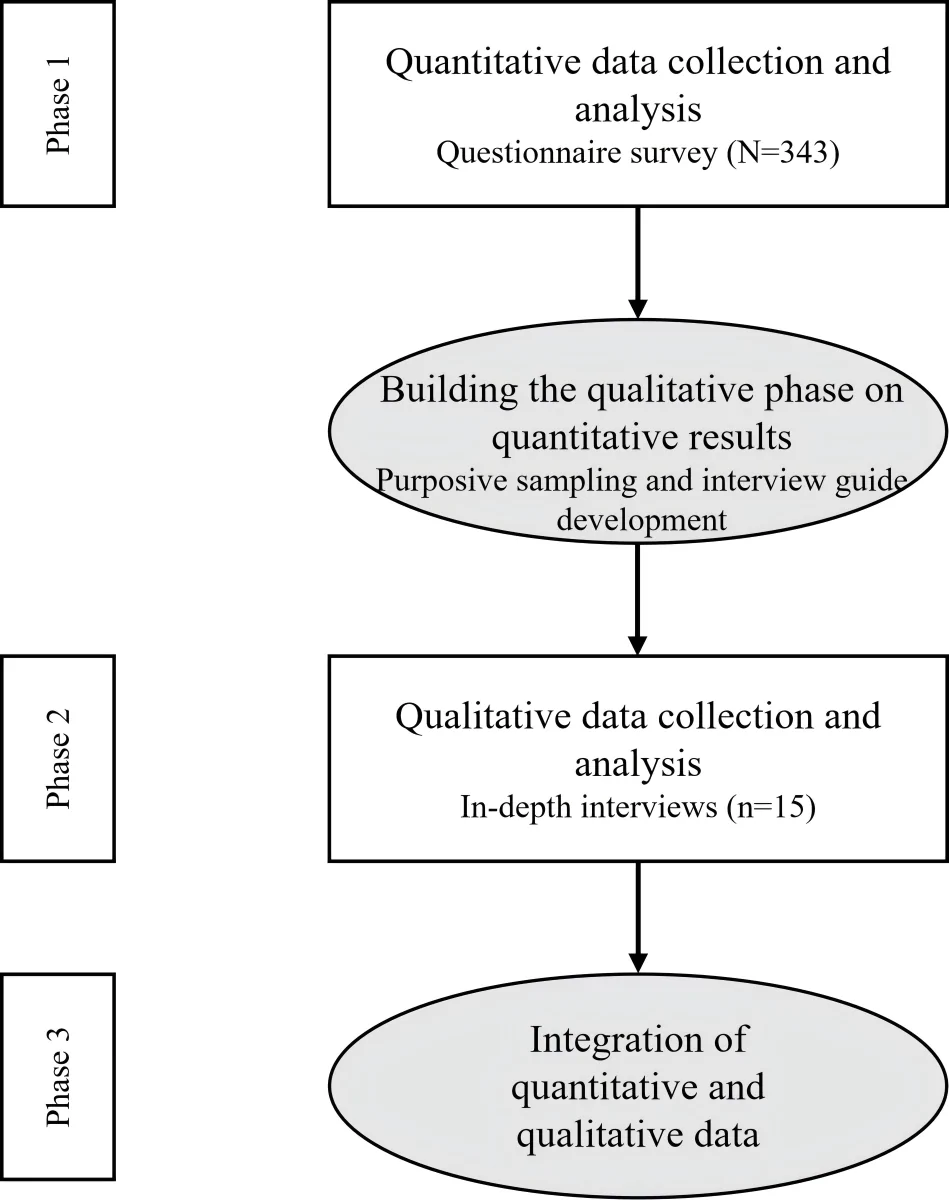
Flowchart for the explanatory sequential mixed methods design.

This study used an explanatory sequential mixed methods design [38]. The overall study flow is presented in Figure 1. Quantitative data were first collected via a cross-sectional questionnaire survey. Participants for the qualitative phase were drawn from the quantitative sample and selected purposively based on the survey results. Semistructured interviews were then conducted. Similar mixed methods approaches have previously been used to investigate the adoption and acceptance of emerging technologies [39,40].

The reporting of this mixed methods study was guided by the GRAMMS (Good Reporting of A Mixed Methods Study) checklist [41], the quantitative cross-sectional component was reported in accordance with the STROBE (Strengthening the Reporting of Observational Studies in Epidemiology) checklist [42], and the qualitative component was reported in accordance with the COREQ (Consolidated Criteria for Reporting Qualitative Research) checklist [43]. The completed checklists are provided in Multimedia Appendix 1.

### Quantitative Phase

#### Inclusion Criteria and Recruitment

Between January 15, 2024, and January 2, 2025, older patients with stroke were recruited using convenience sampling from the Department of Neurology of a tertiary grade A hospital in Shanghai, China. Potential participants were initially identified by trained researchers according to the clinical diagnoses documented in the medical records and were then screened against the inclusion and exclusion criteria. Patients who met the eligibility criteria were approached face-to-face by the researchers and were informed of the study purpose, requirements for questionnaire completion, data confidentiality, and their right to voluntary participation. Written informed consent was obtained before enrollment. The inclusion criteria were as follows: (1) meeting the diagnostic criteria for stroke as defined in the China Stroke Report 2020 [44], (2) aged 60-100 years, and (3) having adequate language expression and communication abilities, with sufficient understanding to cooperate with the study procedures. The exclusion criteria were as follows: (1) a history of psychiatric illness or severe cognitive impairment; (2) the presence of other major organ diseases involving the heart, liver, or kidneys; and (3) current participation in other studies.

Multiple linear regression was used as the primary analytic method. The sample size was estimated using the rule of thumb approach for regression analysis, based on 10-15 participants per candidate predictor. A total of 29 candidate independent variables were planned for inclusion in the model. Allowing for 10% invalid questionnaires, the estimated required sample size ranged from 323 to 484 participants.

#### Variables and Measures

The general information questionnaire was developed by the research team based on the study objectives, relevant literature, and the context of digital health technology use among older patients with stroke. The questionnaire comprised 18 items covering sociodemographic characteristics, disease-related characteristics, and patterns of digital health technology use. Sociodemographic variables included age, sex, marital status, educational level, employment status, monthly per capita household income, and type of medical coverage. Disease-related variables included stroke subtype, duration since stroke onset, number of stroke episodes, the Barthel Index, the modified Rankin Scale (mRS), and current self-rated health status. Variables related to digital health technology use included intention to use digital health technologies, frequency of digital health technology use, use of health-related mobile apps, use of health-related online media, and use of home-based health management devices. To ensure the content relevance and comprehensibility of the study-specific items, the initial draft was reviewed by experts in neurological nursing, rehabilitation nursing, and digital health research, and minor wording revisions were made in accordance with their recommendations. Before the formal survey, the full survey package was pilot tested with a small group of older patients with stroke who met the inclusion criteria to assess item clarity, response burden, and feasibility of completion. These individuals participated only in the pilot test and were not included in the formal quantitative dataset or any subsequent analyses. The pilot test indicated that the study-specific items were generally clear and understandable, and no changes were made to the item content or scoring procedures of the established scales used in this study.

Technophobia was assessed using the Chinese version of the TS. The scale comprises 3 dimensions, namely, technology tension, technology fear, and privacy and security concerns, with a total score ranging from 13 to 65. Higher scores indicate higher levels of technophobia [11,45]. No validated cutoff score was specified for the Chinese TS used in this study; therefore, the total score was treated as a continuous measure. eHealth literacy was assessed using the Chinese version of the eHealth Literacy Scale (eHEALS). This scale includes 3 dimensions, namely, the ability to apply, appraise, and make decisions regarding online health information and services, with a total score ranging from 8 to 40. Higher scores indicate higher levels of eHealth literacy [46,47]. Perceived social support was assessed using the Chinese version of the Perceived Social Support Scale (PSSS). This scale includes 3 dimensions, namely, family support, friend support, and other support, comprising a total of 12 items, with a total score ranging from 12 to 84. Higher scores indicate higher levels of perceived social support [48,49]. Stroke self-efficacy was assessed using the Chinese version of the Stroke Self-Efficacy Questionnaire (SSEQ). The SSEQ was selected because it assesses patients’ confidence in performing poststroke activities of daily living and undertaking stroke self-management. These domains may be relevant to the use of digital health technologies by older patients with stroke for follow-up care, rehabilitation, health monitoring, and self-management; however, the SSEQ does not specifically assess self-efficacy for digital health technology use. The scale comprises 11 items across 2 dimensions: self-efficacy in activities of daily living and self-management efficacy. Total scores range from 0 to 110, with higher scores indicating higher levels of stroke self-efficacy [50,51]. In the present sample, the Cronbach α coefficients for the TS, eHEALS, PSSS, and SSEQ were 0.901, 0.933, 0.940, and 0.911, respectively, indicating good internal consistency [52]. For the TS, confirmatory factor analysis supported the prespecified 3-factor structure, with *χ*²_62_=144.9, *χ*²/df=2.337, comparative fit index=0.981, Tucker-Lewis index=0.976, root mean square error of approximation=0.062, and standardized root mean square residual=0.041, indicating acceptable model fit according to commonly used criteria [53].

### Data Collection

Quantitative data were collected using a paper-based questionnaire administered by trained researchers. After enrollment, participants completed the questionnaire independently whenever possible. For patients who were unable to complete the questionnaire independently because of poststroke upper limb dysfunction, difficulty writing, or fatigue, but who were able to understand the questionnaire content and communicate their response choices verbally, data were collected using a standardized researcher-assisted procedure. A trained researcher (YY) read each item and its response options aloud and recorded the answers selected by the patient without interpreting the items, suggesting answers, or otherwise influencing the responses. Family members or caregivers did not complete any questionnaire items on behalf of the patients. Each returned questionnaire was reviewed on-site for completeness, and participants were invited to complete omitted items where feasible. Questionnaires with more than 10% of items remaining unanswered after this review were excluded from the analysis. Throughout data collection, questionnaire data were entered and checked for accuracy by the research team in weekly batches.

### Statistical Analysis

To ensure data entry accuracy, data from the paper-based questionnaires were independently entered into an EpiData 3.0 database by 2 researchers (YY and YoW). Before applying the questionnaire-level exclusion criterion, missingness patterns were characterized descriptively by summarizing the number and proportion of unanswered items per questionnaire and examining their distribution across individual items, scales, and questionnaire sections. No missing value imputation was performed. Statistical analyses were performed using SPSS (version 27.0; IBM Corp). All statistical tests were 2-sided, with *P*<.05 indicating statistical significance. Categorical variables were summarized as frequencies and percentages. Continuous variables were assessed for normality using the Shapiro-Wilk test and summarized as mean (SD) when approximately normally distributed or as median (IQR) otherwise. Univariable analyses were conducted to compare technophobia scores across participant-characteristic subgroups. Homogeneity of variance was assessed using the Levene test. When the assumptions of approximate normality and homogeneity of variance were met, independent-samples *t* tests were used for variables with 2 categories and 1-way ANOVA for variables with more than 2 categories. Otherwise, the Mann-Whitney *U* test or Kruskal-Wallis *H* test was used, as appropriate.

Correlation analyses were conducted to examine the associations of continuous and ordinal variables with technophobia. Pearson correlation analysis was used for approximately normally distributed continuous variables, whereas Spearman rank correlation analysis was used for ordinal variables or continuous variables that departed from normality. The correlation matrix heatmap was generated using GraphPad Prism (version 10.6; GraphPad Software).

A multivariable linear regression model was fitted with the total technophobia score as the dependent variable. Variables associated with technophobia at *P*≤.10 in either the univariable or correlation analyses were entered simultaneously into the model using the enter method. Categories for the independent variables were prespecified based on previous literature. Categorical variables were dummy coded, with the lowest category used as the reference for ordered variables to permit comparisons with higher categories; continuous variables were entered on their original scales. The complete coding scheme is provided in Multimedia Appendix 2. Residual normality was assessed using the histogram and normal P-P plot of standardized residuals, and homoscedasticity was evaluated using the scatterplot of standardized residuals against standardized predicted values. Multicollinearity was assessed using tolerance values and variance inflation factors.

### Qualitative Data: Inclusion Criteria and Recruitment

Participants in the qualitative phase were selected from older patients with stroke who had completed the quantitative survey and had agreed during the quantitative phase to be contacted for a subsequent interview. In addition, participants were required to be able to describe their experiences of using or avoiding digital health technologies and to be willing to participate in a semistructured interview. A maximum variation purposive sampling strategy was used. Using participant-level data from the quantitative phase, potential interviewees were selected to span the observed distribution of total technophobia scores, while variation in sex, age, and educational attainment was also sought. Researchers contacted potential participants either face-to-face or by telephone to confirm their willingness to participate and to arrange a suitable interview time and location.

### Data Collection and Procedures

Between February 6 and April 30, 2025, semistructured interviews were conducted by 2 female master’s students in nursing who had received formal training in qualitative research. All interviews were conducted individually and face-to-face in a quiet, private office at the study hospital, with no family members, caregivers, clinical staff, or other nonparticipants present. Both interviewers were members of the research team and were familiar with the context of stroke care and rehabilitation, but neither was involved in participants’ direct clinical treatment, nursing care, or care-related decision-making. Before each interview, the interviewers introduced themselves as researchers and explained the study purpose, audio-recording arrangements, confidentiality safeguards, the right to withdraw, and that participation or nonparticipation would not affect the care participants received. Written informed consent was then obtained. With participants’ permission, all interviews were audio-recorded, and no repeat interviews were conducted. After each interview, the interviewers recorded reflexive notes documenting the interview context, participants’ nonverbal cues, and their reflections on the interview interaction. During research team discussions, they critically considered how their professional backgrounds, researcher roles, and interactions with participants might have shaped data generation and interpretation.

Interviews were scheduled when participants’ health status permitted and sufficient time was available. The semistructured interview guide was jointly developed by the research team on the basis of the quantitative-phase findings and relevant literature, with the quantitative findings also used to identify topics requiring further explanatory probing. The guide was reviewed by the principal investigator (YL), who had formal training and experience in qualitative research. Before formal data collection, the guide was pilot-tested with 2 patients who met the eligibility criteria. Data from the pilot interviews were not included in the formal analysis, and the wording of selected questions and follow-up prompts were refined in response to participant feedback. The semistructured interview guide covered five areas: (1) experiences of using digital health technologies and their perceived role in health care, rehabilitation, and health self-management; (2) tension, concerns, and difficulties encountered during use; (3) understanding and evaluating online health information, confidence in completing related tasks, and responses to difficulties; (4) contextual conditions, including social support, device and internet access, costs, and poststroke functional changes; and (5) suggestions for improving digital health technologies and services delivered through them. Questions were phrased in plain, open-ended, and nonjudgmental language and were sequenced from general topics to specific experiences, allowing participants to describe their experiences at their own pace. If a participant showed a marked emotional reaction, the interviewer paused discussion of the current topic or moved to another topic to respect the participant’s wishes and minimize discomfort. Each interview lasted 20-45 minutes. The complete interview guide is provided in Multimedia Appendix 3.

Qualitative data collection and preliminary analysis proceeded concurrently. After each interview, the research team conducted a preliminary review and discussion of the interview transcript and reflexive notes to assess whether the accumulating data continued to broaden or deepen the experiences represented or provided additional explanations relevant to the quantitative findings. Data adequacy was considered to have been reached when successive interviews no longer substantially extended the experiences represented in the data or contributed new explanatory information. This point was first judged to have been reached after the 13th interview. Two additional participants were subsequently interviewed to assess whether further interviews would extend the experiential or explanatory scope of the data. These additional interviews added detail to the existing accounts but did not materially broaden the experiences or explanations already represented. The final qualitative sample therefore comprised 15 participants.

### Analysis

Interview data were analyzed using reflexive thematic analysis as described by Braun and Clarke [54,55]. All interview recordings were transcribed verbatim within 24 hours of each interview and checked against the original audio recordings for accuracy and completeness. The transcripts were then imported into NVivo (version 12.0; QSR International Pty Ltd) for data management.

Two researchers (YY and YoW) repeatedly read all interview transcripts to become familiar with the dataset and independently generated initial codes. At successive stages of coding and theme development, they compared their coding decisions, analytic reflections, and interpretations of the data. When different interpretations arose regarding the meaning, boundaries, or organization of codes or themes, the 2 researchers first returned to the relevant interview extracts and considered each interpretation in relation to the research question and the dataset as a whole. Interpretive differences that could not be resolved through this initial discussion were discussed within the 5-member qualitative research team, which also reviewed the developing thematic structure. During these discussions, the team collectively examined the relevant data extracts, their broader context within the interviews, and the analytic rationale underlying the alternative interpretations. The corresponding codes or themes were revised, merged, separated, or renamed through discussion until consensus was reached. This collaborative process was used to enrich interpretation through reflexive dialogue rather than to establish intercoder reliability.

The analysis combined deductive and inductive elements: the quantitative findings served as sensitizing context for areas requiring further explanation, but no fixed coding framework or themes were specified in advance. The analysis proceeded recursively through familiarization with the data, generating initial codes, generating initial themes, reviewing potential themes, defining and naming themes, and producing the report. Candidate themes were reviewed in relation to the coded extracts and the full dataset to assess their coherence, distinctiveness, and relevance to the research question. Coding and theme development were treated as iterative and interpretive processes grounded in sustained engagement with the data. The research team met regularly to examine emerging interpretations and reflect on how analytic assumptions shaped the analysis. YL provided methodological oversight throughout the qualitative analysis and reviewed the final thematic structure.

All interviews were conducted in Chinese, and transcription, coding, and theme development were also undertaken in Chinese. The full interview transcripts were not translated into English. After completion of the qualitative analysis, the finalized theme names and narrative descriptions of the qualitative findings were translated into English for reporting in the manuscript. Selected participant quotations used to illustrate the themes were also translated into English. Translation was performed by a professional translator assigned by an independent translation company. The translator was a native English speaker proficient in Chinese. YL and YY verified each translated component against the original Chinese analytic materials and interview transcripts and subsequently reviewed the translations with the translator to ensure semantic accuracy, contextual fidelity, consistency with the original analysis, and preservation of the participants’ intended meanings. Any discrepancies in wording or interpretation were resolved through discussion with reference to the original Chinese materials. No generative AI or machine translation tools were used at any stage of the translation or verification process.

### Mixed Methods Data Integration

Integration was undertaken at 3 stages, following established guidance for mixed methods integration [56]. The mixed methods study flow and integration process are summarized in Figure 1. First, the quantitative findings informed purposive sampling for the qualitative phase and refinement of the semistructured interview guide. Second, the qualitative findings were used to explain and contextualize the quantitative associations identified in the first phase. Third, the quantitative and qualitative strands were compared in the Results section to assess convergence, complementarity, and divergence. During interpretation, the socioecological perspective was used to organize the integrated findings across individual, interpersonal support, and technology use context levels.

### Ethical Considerations

This study was performed in accordance with the Declaration of Helsinki and received ethics approval from the Shanghai University of Traditional Chinese Medicine Ethics Committee (approval number 2024-1-16‐06). All participants were informed of the study objectives and significance, voluntarily signed informed consent forms, and were advised that they could withdraw from the study at any time without providing a reason, without any impact on their medical care or legal rights. The collected data were anonymized or coded and securely stored in accordance with the General Data Protection Regulation guidelines.

## Results

### Quantitative Findings: Participant Characteristics

**Figure 2.**
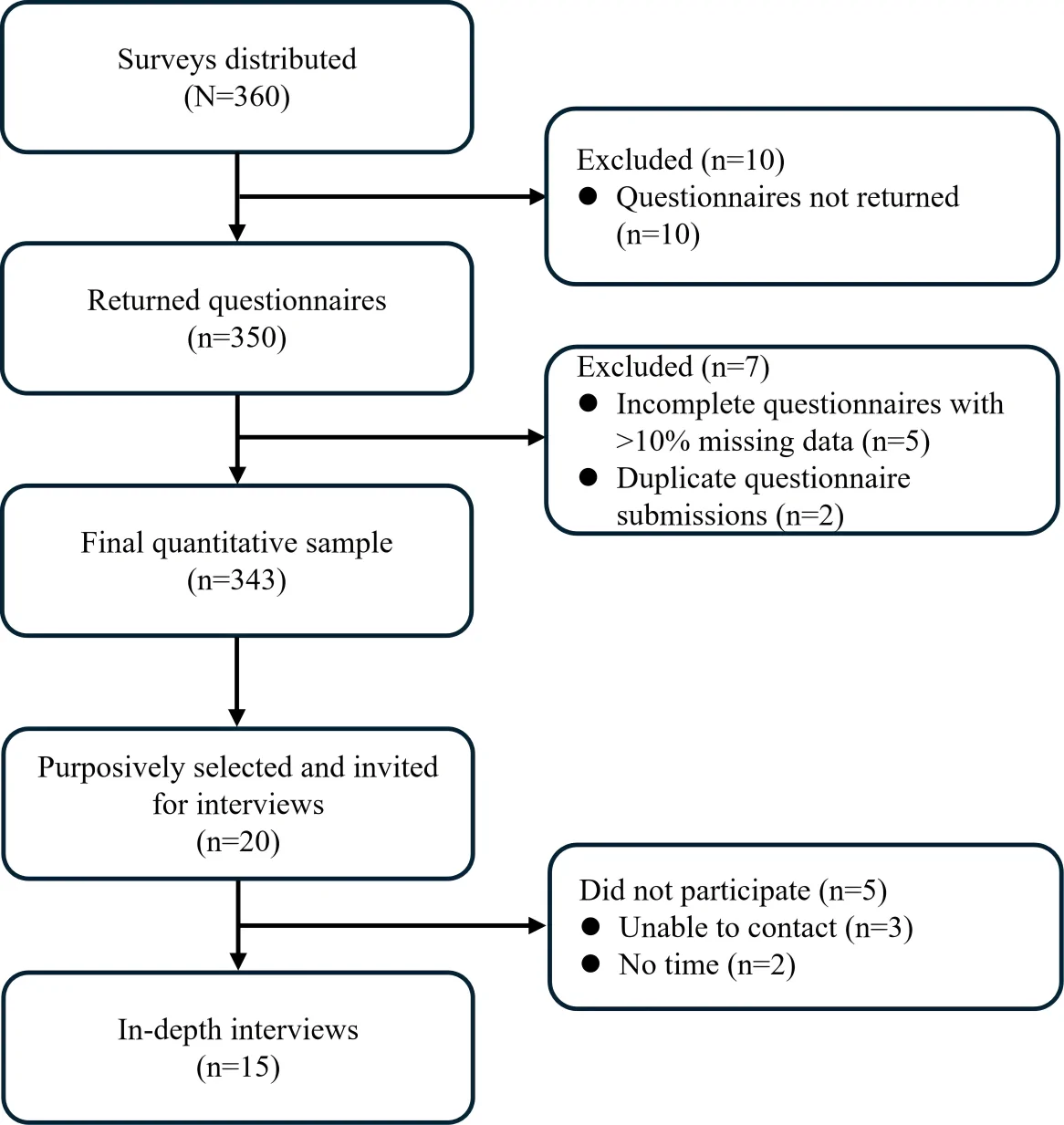
Flow diagram of participant recruitment and study participation.

Of the 360 questionnaires distributed, 350 were returned, whereas 10 were not returned. Seven returned questionnaires were excluded: 5 because more than 10% of the items were unanswered and 2 because they were duplicate submissions. Examination of the 5 incomplete questionnaires indicated that missing responses were dispersed across items, scales, and questionnaire sections, without a consistent concentration in any particular item, scale, or section. Accordingly, descriptive examination did not identify an evident systematic pattern of missingness. After these exclusions, the final quantitative analytic sample comprised 343 participants, with no missing values in the final dataset (Figure 2). Among the 343 participants, the median age was 68.0 (IQR 64.0‐73.0) years, and 58.3% (200/343) were male. Most participants had ischemic stroke (266/343, 77.5%), and the median mRS score was 2.0 (IQR 1.0‐3.0). Regarding digital health technologies, 80.2% (275/343) reported an intention to use digital health technologies, and sometimes use was the most commonly reported frequency (144/343, 42.0%). Detailed participant characteristics are presented in Table 1.

**Table 1.** Participant characteristics and univariable comparisons of total Technophobia Scale scores (N=343).

Characteristics	Values	Total TS^a^ score, median (IQR)	Test statistic	*P* value
Sociodemographic factors				
Age (years), median (IQR)	68.0 (64.0‐73.0)	N/A^b^	N/A	N/A
Sex, n (%)			−0.983^c^	.33
Male	200 (58.3)	25.0 (22.3‐33.0)		
Female	143 (41.7)	25.0 (22.0‐34.0)		
Marital status, n (%)			0.377^d^	.95
Never married	9 (2.6)	28.0 (23.0‐29.0)		
Married	288 (84)	25.0 (22.0‐33.8)		
Divorced	9 (2.6)	26.0 (22.5‐32.5)		
Widowed	37 (10.8)	25.0 (20.5‐36.0)		
Education, n (%)			−2.741^c^	.006^e^
High school or below	242 (70.6)	26.0 (23.0‐35.0)		
Higher education or above	101 (29.4)	24.0 (21.0‐29.5)		
Employment status, n (%)			6.859^d^	.03^e^
Employed	21 (6.1)	28.0 (25.0‐31.0)		
On sick leave or retired	301 (87.8)	25.0 (22.0‐33.0)		
Unemployed	21 (6.1)	30.0 (24.0‐38.5)		
Monthly per capita household income (RMB^f^), n (%)			15.942^d^	.007^e^
<1000^f^	23 (6.7)	25.0 (22.0‐47.0)		
1000‐2999	61 (17.8)	28.0 (23.5‐40.0)		
3000‐4999	118 (34.4)	27.0 (23.0‐36.0)		
5000‐6999	102 (29.7)	24.0 (21.0‐28.3)		
7000‐9999	26 (7.6)	25.0 (21.8‐30.0)		
≥10,000	13 (3.8)	25.0 (21.0‐31.5)		
Type of medical coverage, n (%)			5.324^d^	.07
Self-pay	17 (5)	25.0 (23.0‐38.5)		
Urban employee medical insurance	270 (78.7)	25.0 (22.0‐33.0)		
New rural cooperative medical scheme	56 (16.3)	28.0 (24.3‐35.8)		
Disease-related characteristics				
Barthel Index score, median (IQR)	75.0 (50.0‐85.0)	N/A	N/A	N/A
Stroke subtype, n (%)			0.307^d^	.86
Hemorrhagic stroke	61 (17.8)	25.0 (23.0‐34.5)		
Ischemic stroke	266 (77.5)	25.0 (22.0‐33.0)		
Mixed stroke	16 (4.7)	24.5 (22.0‐41.0)		
Duration since stroke onset (months), n (%)			3.491^d^	.32
<3	86 (25.1)	27.5 (23.8‐33.0)		
3‐6	59 (17.2)	25.0 (21.0‐33.0)		
7‐12	76 (22.1)	24.0 (21.0‐34.8)		
>12	122 (35.6)	25.0 (22.0‐35.3)		
Number of stroke episodes, n (%)			7.055^d^	.0^e^3
1	246 (71.7)	25.0 (22.0‐32.0)		
2	67 (19.5)	26.0 (23.0‐34.0)		
≥3	30 (8.7)	32.0 (24.0‐43.3)		
Current self-rated health status, n (%)			9.418^d^	.009^e^
Poor	180 (52.5)	24.0 (22.0‐32.8)		
Fair	101 (29.4)	27.0 (23.0‐35.0)		
Good	62 (18.1)	28.5 (23.0‐34.0)		
Modified Rankin Scale score, n (%)			9.537^d^	.008^e^
0‐1	90 (26.2)	28.0 (23.0‐33.0)		
2‐3	189 (55.1)	24.0 (22.0‐32.0)		
≥4	64 (18.7)	26.5 (23.0‐36.8)		
Use of digital health technologies				
Intention to use digital health technologies, n (%)			−3.860^c^	<.001^e^
Yes	275 (80.2)	24.0 (22.0‐31.0)		
No	68 (19.8)	32.0 (25.0‐38.0)		
Frequency of digital health technology use, n (%)			15.033^d^	.002^e^
Never	36 (10.5)	32.0 (24.3‐42.8)		
Occasionally	126 (36.7)	28.0 (23.0‐35.3)		
Sometimes	144 (42)	24.0 (21.0‐29.5)		
Often	37 (10.8)	25.0 (23.0‐29.5)		
Use of health-related mobile apps, n (%)			−1.216^c^	.22
Yes	88 (25.7)	24.0 (21.0‐38.0)		
No	255 (74.3)	26.0 (22.0‐33.0)		
Use of health-related online media, n (%)			−3.677^c^	<.001^e^
Yes	167 (48.7)	24.0 (22.0‐29.0)		
No	176 (51.3)	28.0 (23.0‐37.8)		
Use of home health management devices, n (%)			−3.292^c^	<.001^e^
Yes	198 (57.7)	27.5 (23.0‐35.3)		
No	145 (42.3)	24.0 (22.0‐29.0)		

a TS: Technophobia Scale.

b Not applicable.

c Z statistic (Mann-Whitney *U* test).

d H statistic (Kruskal-Wallis H test).

e Statistically significant (*P*<.05).

f RMB: Renminbi (Chinese yuan); RMB 1=US $0.14 as of January 29, 2026.

### Current Status of Technophobia Toward Digital Health Technologies Among Older Patients With Stroke

Data analysis revealed that the median TS total score among older patients with stroke was 25.0 (IQR 22.0‐33.0). The median scores for the subscales were 10.0 (IQR 8.0‐17.0) for technology tension, 10.0 (IQR 8.0‐12.0) for technology fear, and 6.0 (IQR 5.0‐8.0) for privacy and security concerns (Table 2).

**Table 2.** Total and subscale scores of the Technophobia Scale among older patients with stroke (N=343).

Variable	Items, n	Possible range	Observed range	Total score^a^		Mean item score^a^	
				Median (IQR)	Mean (SD)	Median (IQR)	Mean (SD)
Total technophobia score	13	13‐65	17‐59	25.0 (22.0‐33.0)	29.5 (10.3)	1.9 (1.7‐2.5)	2.3 (0.8)
Technology tension	5	5‐25	5‐25	10.0 (8.0‐17.0)	12.0 (5.2)	2.0 (1.6‐3.4)	2.4 (1.0)
Technology fear	5	5‐25	5‐23	10.0 (8.0‐12.0)	10.6 (4.0)	2.0 (1.6‐2.4)	2.1 (0.8)
Privacy and security concerns	3	3‐15	3‐15	6.0 (5.0‐8.0)	6.9 (3.2)	2.0 (1.7‐2.7)	2.3 (1.1)

a Scale scores are presented as medians (IQR) due to nonnormal distributions; the mean (SD) is also reported to facilitate comparisons with previous studies.

### Scores of Variables Associated With Technophobia Toward Digital Health Technologies Among Older Patients With Stroke

Descriptive statistics were used to summarize factors associated with technophobia toward digital health technologies among older patients with stroke. The median eHealth literacy score was 30.0 (IQR 18.0‐34.0), the median perceived social support score was 66.0 (IQR 52.0‐72.0), and the median stroke self-efficacy score was 83.0 (IQR 64.0‐89.0). Detailed results are presented in Table 3.

**Table 3.** Scores of variables associated with technophobia toward digital health technologies among older patients with stroke (N=343).

Variable	Items, n	Possible range	Observed range	Total score^a^		Mean item score^a^	
Median (IQR)	Mean (SD)	Median (IQR)	Mean (SD)
Total eHealth literacy score	8	8‐40	9‐39	30.0 (18.0‐34.0)	26.5 (8.7)	3.8 (2.3‐4.3)	3.3 (1.1)
Use ability	5	5‐25	5‐25	19.0 (10.0‐22.0)	16.6 (5.9)	3.8 (2.0‐4.4)	3.3 (1.2)
Appraisal ability	2	2‐10	2‐10	8.0 (5.0‐9.0)	6.8 (2.4)	4.0 (2.5‐4.5)	3.4 (1.2)
Decision-making ability	1	1‐5	1‐5	3.0 (2.0‐4.0)	3.2 (1.3)	3.0 (2.0‐4.0)	3.2 (1.3)
Total perceived social support score	12	12‐84	24‐77	66.0 (52.0‐72.0)	60.2 (14.3)	5.5 (4.3‐6.0)	5.0 (1.2)
Family support	4	4‐28	6‐28	24.0 (22.0‐25.0)	22.2 (5.2)	6.0 (5.5‐6.3)	5.6 (1.3)
Friend support	4	4‐28	7‐28	21.0 (15.0‐24.0)	19.3 (5.3)	5.3 (3.8‐6.0)	4.8 (1.3)
Other support	4	4‐28	5‐27	20.0 (14.0‐24.0)	18.7 (5.8)	5.0 (3.5‐6.0)	4.7 (1.5)
Total stroke self-efficacy score	11	0‐110	20‐101	83.0 (64.0‐89.0)	75.8 (17.5)	7.6 (5.8‐8.1)	6.9 (1.6)
Activity function efficacy	6	0‐60	7‐60	45.0 (36.0‐49.0)	41.2 (11.6)	7.5 (6.0‐8.2)	6.9 (1.9)
Self-management efficacy	5	0‐50	8‐47	36.0 (30.0‐40.0)	34.6 (7.6)	7.2 (6.0‐8.0)	6.9 (1.5)

a Scale scores are presented as medians (IQR) due to nonnormal distributions; the mean (SD) is also reported to facilitate comparisons with previous studies.

### Univariable Analysis

Univariable analyses showed that total technophobia scores differed significantly by educational level, employment status, monthly per capita household income, mRS score, number of stroke episodes, current self-rated health status, intention to use digital health technologies, frequency of digital health technology use, use of health-related online media, and use of home-based health management devices (*P*<.05; Table 1).

### Correlation Analysis

**Figure 3.**
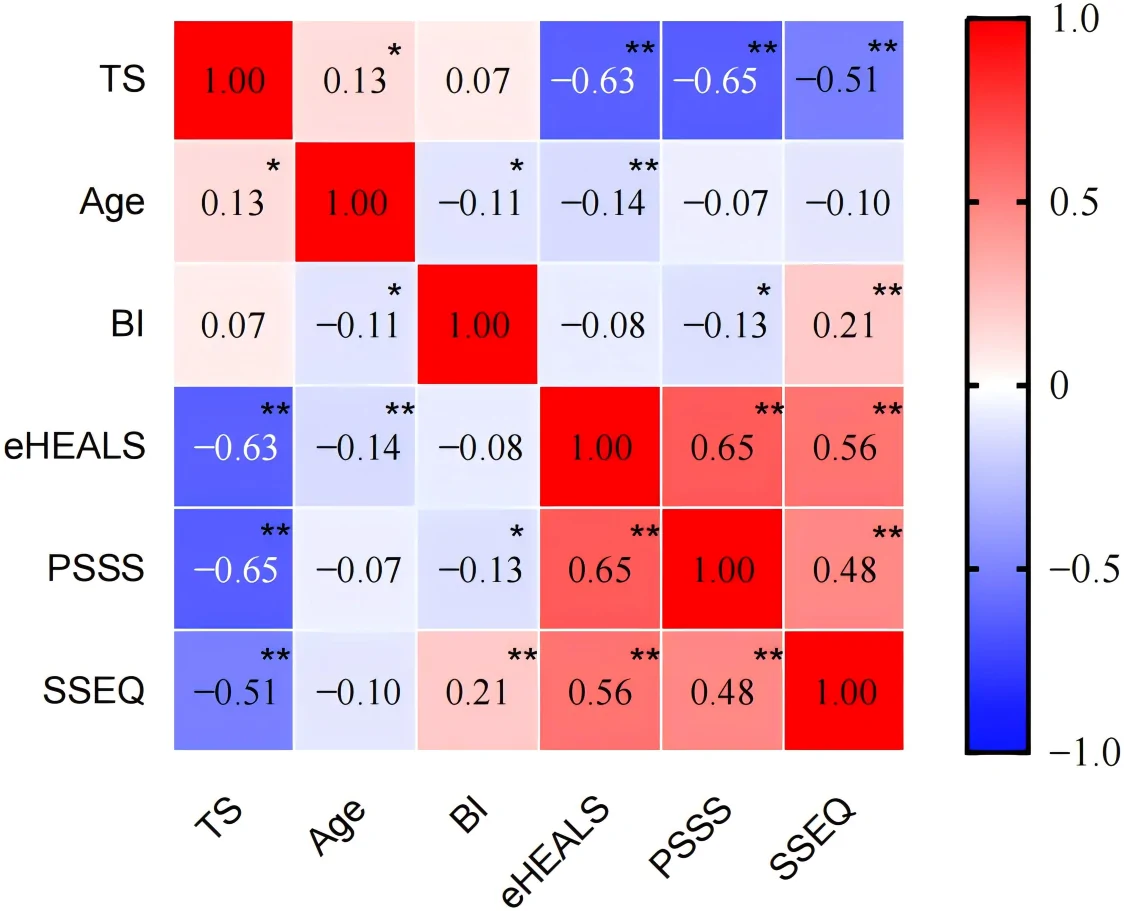
Spearman rank correlation heatmap of technophobia toward digital health technologies and related variables among older patients with stroke. Values within the cells represent Spearman rank correlation coefficients (ρ). BI: Barthel Index; eHEALS: eHealth Literacy Scale; PSSS: Perceived Social Support Scale; SSEQ: Stroke Self-Efficacy Questionnaire; TS: Technophobia Scale. *P<.05; **P<.01; all *P* values were 2-sided.

Spearman correlation analysis showed that technophobia toward digital health technologies among older patients with stroke was positively correlated with age (ρ=0.133; *P*=.01) and negatively correlated with eHealth literacy (ρ=−0.632; *P*<.001), perceived social support (ρ=−0.645; *P*<.001), and stroke self-efficacy (ρ=−0.511; *P*<.001). The detailed correlation coefficients and *P* values are presented in Table 4, and the direction and relative magnitude of the correlations among the study variables are visualized in Figure 3.

**Table 4.** Correlation analysis (Spearman ρ^a^ and *P* value) among the research variables.

Variable	Technophobia score	Age (years)	Barthel Index score	eHealth literacy score	Perceived social support score	Stroke self-efficacy score
Technophobia score						
ρ	1	0.133^b^	0.067	−0.632^b^	−0.645^b^	−0.511^b^
*P* value	N/A^c^	.01	.21	<.001	<.001	<.001
Age (years)						
ρ	0.133^b^	1	−0.107^b^	−0.143^b^	−0.068	−0.099
*P* value	.01	N/A	.047	.008	.21	.07
Barthel Index score						
ρ	0.067	−0.107^b^	1	−0.076	−0.126^b^	0.209^b^
*P* value	.21	.047	N/A	.16	.02	<.001
eHealth literacy score						
ρ	−0.632^b^	−0.143^b^	−0.076	1	0.650^b^	0.557^b^
*P* value	<.001	.008	.16	N/A	<.001	<.001
Perceived social support score						
ρ	−0.645^b^	−0.068	−0.126^b^	0.650^b^	1	0.478^b^
*P* value	<.001	.21	.02	<.001	N/A	<.001
Stroke self-efficacy score						
ρ	−0.511^b^	−0.099	0.209^b^	0.557^b^	0.478^b^	1
*P* value	<.001	.07	<.001	<.001	<.001	N/A

a ρ indicates the Spearman rank correlation coefficient.

b The correlation is significant at a significance level of .05 (2-sided).

c Not applicable.

### Multivariable Linear Regression Analysis

Fifteen variables that were associated with technophobia at *P*≤.10 in the univariable or correlation analyses were entered into the multivariable linear regression model. Residual diagnostics did not indicate substantial departures from residual normality or an obvious funnel-shaped pattern in the plot of standardized residuals against standardized predicted values, suggesting no marked heteroscedasticity. All tolerance values were >0.1, and all variance inflation factors were <10, indicating no evidence of problematic multicollinearity among the independent variables. In the adjusted model, monthly per capita household income, frequency of digital health technology use, eHealth literacy, perceived social support, and stroke self-efficacy were associated with technophobia toward digital health technologies (*P*<.05). Specifically, patients with a monthly per capita household income of Renminbi (Chinese yuan; RMB) 5000‐6999 (RMB 1=US $0.14 as of January 29, 2026) had lower technophobia scores than those with an income of less than RMB 1000 (*B*=−3.60, 95% CI −6.44 to −0.76). Compared with patients who never used digital health technologies, those who used them occasionally also had lower technophobia scores (*B*=−3.98, 95% CI −6.94 to −1.03). Higher eHealth literacy (*B*=−0.45, 95% CI −0.58 to −0.32), perceived social support (*B*=−0.35, 95% CI −0.42 to −0.29), and stroke self-efficacy (*B*=−0.08, 95% CI −0.13 to −0.02) were associated with lower technophobia scores. Detailed results for the statistically significant variables are presented in Table 5, and the diagnostic plots and complete multivariable linear regression results are provided in Multimedia Appendix 2.

**Table 5.** Significant variables associated with technophobia toward digital health technologies among older patients with stroke in the multivariable linear regression model (N=343)^a^. Only variables statistically significant at *P*<.05 are shown. The complete multivariable linear regression results are provided in Multimedia Appendix 2.

Variable	*B* ^b^	SE	β^c^	*t* test (*df*)	95% CI	*P* value
Intercept	75.01	5.04	N/A^d^	14.89 (316)	65.09 to 84.92	<.001
Monthly per capita household income (RMB^e^)						
5000–6999^e^	−3.60	1.44	−0.16	−2.49 (316)	−6.44 to −0.76	.01
Frequency of digital health technology use						
Occasionally	−3.98	1.50	−0.19	−2.65 (316)	−6.94 to −1.03	.008
eHealth literacy	−0.45	0.07	−0.38	−6.82 (316)	−0.58 to −0.32	<.001
Perceived social support	−0.35	0.03	−0.49	−10.63 (316)	−0.42 to −0.29	<.001
Stroke self-efficacy	−0.08	0.03	−0.13	−2.60 (316)	−0.13 to −0.02	.01

a *R*=0.83, *R²*=0.69, adjusted *R²*=0.66, *F*=26.87, *P*<.001.

b *B* indicates unstandardized regression coefficient.

c β indicates standardized regression coefficient.

d N/A: not applicable.

e RMB: Renminbi (Chinese yuan); RMB 1=US $0.14 as of January 29, 2026.

### Qualitative Findings

#### Participant Characteristics

Fifteen participants were interviewed. Their median age was 69.0 (IQR 65.0‐74.0) years, and 53.3% (8/15) were male. Ischemic stroke was the predominant subtype, accounting for 66.7% (10/15) of participants. The median technophobia score was 29.0 (IQR 22.0‐33.0). Detailed participant characteristics are provided in Multimedia Appendix 4. The analysis generated 4 themes, 2 of which comprised 2 subthemes each. An overview of the themes and subthemes is presented in Table 6.

**Table 6.** Qualitative themes, subthemes, and analytic focus.

Theme	Subtheme	Analytic focus
Affordability of Digital Health Technologies	Not further subdivided	Financial pressure and fear that operational errors could result in additional out-of-pocket costs
Adoption and Proficiency in Using Digital Health Technologies	Not further subdivided	Greater familiarity and trust through repeated use, together with willingness to adopt AI-assisted approaches to health management
Influence of Social Relationship Networks	Support From Family and Friends	Practical assistance, clarification, and reassurance from family members and friends, accompanied by reduced nervousness and greater social connectedness
Influence of Social Relationship Networks	Insufficient Support	Strong willingness to use digital health technologies despite limited access to guidance, operational difficulties, distress, and frustration
Resources for Digital Health Technology Competence	Understanding and Appraising Online Health Information	Difficulty appraising online health information, together with concerns about fraudulent content, privacy, and security
Resources for Digital Health Technology Competence	Active Exploration and Sustained Use	Confidence in learning, continued attempts, and sustained engagement when difficulties were encountered

#### Affordability of Digital Health Technologies

Participants’ accounts suggested that affordability influenced technophobia toward digital health technologies by shaping how they appraised the potential consequences of operational errors. For patients already facing substantial treatment and rehabilitation expenses, an operational error was perceived not merely as a minor inconvenience but as a potential source of additional financial burden. The anticipated risk of incurring unexpected charges appeared to heighten their vigilance, hesitation, and fear when using digital health technologies.

*...Since I got sick, every follow-up costs money, such as tests, appointments, and all of it. Treatment and rehab have already put me under a lot of financial pressure, so I have to stretch every penny. That’s why whenever I use anything medical online, I get a little scared and extra careful. I’m afraid I’ll hit the wrong thing by accident and end up getting charged for no reason...*

[Male, aged 65 years]

#### Adoption and Proficiency in Using Digital Health Technologies

Interview findings suggested that repeated engagement with digital health technologies reduced uncertainty by transforming initially unfamiliar tasks into increasingly familiar routines. Successfully retrieving health information, scheduling medical appointments, and accessing test results strengthened participants’ familiarity with and trust in these technologies, making subsequent difficulties seem more manageable. Notably, some participants expressed a clear willingness to adopt AI-assisted approaches to health management and described an open and positive stance toward their use.

*...I use WeChat official accounts a lot to look up stuff about stroke. Some of them are really good. I’ve also learned how to book an appointment through the hospital’s official account and check my test results. I can even see the charges from when I was in the hospital... The more you use it, the more you get the hang of it... And lately I’ve started trying AI to answer some of my questions. That AI search feels more thorough than when I look things up myself, and the suggestions are a bit more to the point*.

[Male, aged 61 years]

#### Influence of Social Relationship Networks

Participants’ accounts indicated that social relationships shaped how they experienced and managed technophobia by providing, or failing to provide, practical and emotional resources during technology use.

##### Support From Family and Friends

Interview findings suggested that advice, step-by-step guidance, and timely assistance from family members and friends were experienced as an important form of social support, helping participants navigate operational difficulties when using digital health technologies. The presence of a trusted helper created a sense of security by allowing errors to be identified and corrected promptly, which helped alleviate fear during use. Together, instrumental and emotional support strengthened participants’ confidence in using digital health technologies. Sharing health-related information also facilitated clarification and feedback and strengthened participants’ sense of connection with others.

*Just having someone who really gets it standing next to you makes you feel a lot more at ease. Even if you hit the wrong button or the page gets stuck, they can check it right away and tell you what to do next. And then, using these new things doesn’t feel nearly as scary...*

[Female, aged 65 years]

*I’ve now added several other stroke patients on WeChat. Most days, we just chat in the group about how we’re doing and how our recovery’s been lately. If someone has something new to share or a good tip, they’ll share it with everyone. When there are features I don’t really understand, I’ll turn around and ask my kids. They’re young, so they know this stuff, and little by little I’ve picked up some things too. Back and forth like that, it feels like I’m in touch with people way more than before...*

[Male, aged 71 years]

##### Insufficient Support

In contrast, some participants expressed a strong willingness to use digital health technologies; however, limited access to guidance and support made them more likely to experience practical difficulties during use, often accompanied by pronounced distress and frustration.

*I want to use it, and I want to learn, but there’s no one to teach me. My kids are all busy, and whenever I’m in the hospital, I’m tied up with rehab, so I don’t really have time to ask the doctors or nurses. I don’t have time to learn from other patients either, or just talk things through. No one shows me, and some of it I honestly can’t make sense of at all—I can’t learn it. And it really stresses me out...*

[Male, aged 78 years]

### Resources for Digital Health Technology Competence

Participants’ accounts suggested that competence in using digital health technologies was underpinned by both the ability to appraise online information and the confidence to persist when difficulties arose. Together, these resources appeared to shape whether uncertainty was experienced as an unmanageable threat or as a challenge that could be addressed through continued effort.

#### Understanding and Appraising Online Health Information

Interview findings suggested that participants with limited capacity to appraise online health information had difficulty distinguishing credible content from misleading or fraudulent information. The uncertainty arising from this difficulty appeared to heighten fears of being deceived and concerns about privacy and security, thereby diminishing their willingness to use digital health technologies and creating a substantial barrier to use.

*Once my arm on the weak side started feeling numb, I went online to see what it might be. I wasn’t really paying attention and clicked a pop-up by mistake, and that afternoon someone called me asking if I wanted to buy some ‘special stroke medicine.’ It felt like the moment I went online, my number got out. Since then, I haven’t dared to just use those online consultations anymore*.

[Female, aged 80 years]

#### Active Exploration and Sustained Use

Some participants expressed confidence in their ability to learn and use digital health technologies. This confidence appeared to sustain their efforts when difficulties arose and enabled them to approach technology-related challenges with greater composure. Repeated successful use further reinforced their belief that digital health technologies could be learned and incorporated into rehabilitation and health self-management, thereby reducing apprehension during subsequent use.

*Since I got sick, I’ve been using my phone to learn about stroke. I’ve taken quite a few health education classes and read a lot of information. Through this, I’ve got to know my own condition better and learned how to use these digital tools to help with my daily rehab. Now, when I use them, I feel much more at ease; they don’t seem that complicated anymore. If I take it step by step, I feel I can learn how to use them*.

[Female, aged 72 years]

### Integrated Findings

**Figure 4.**
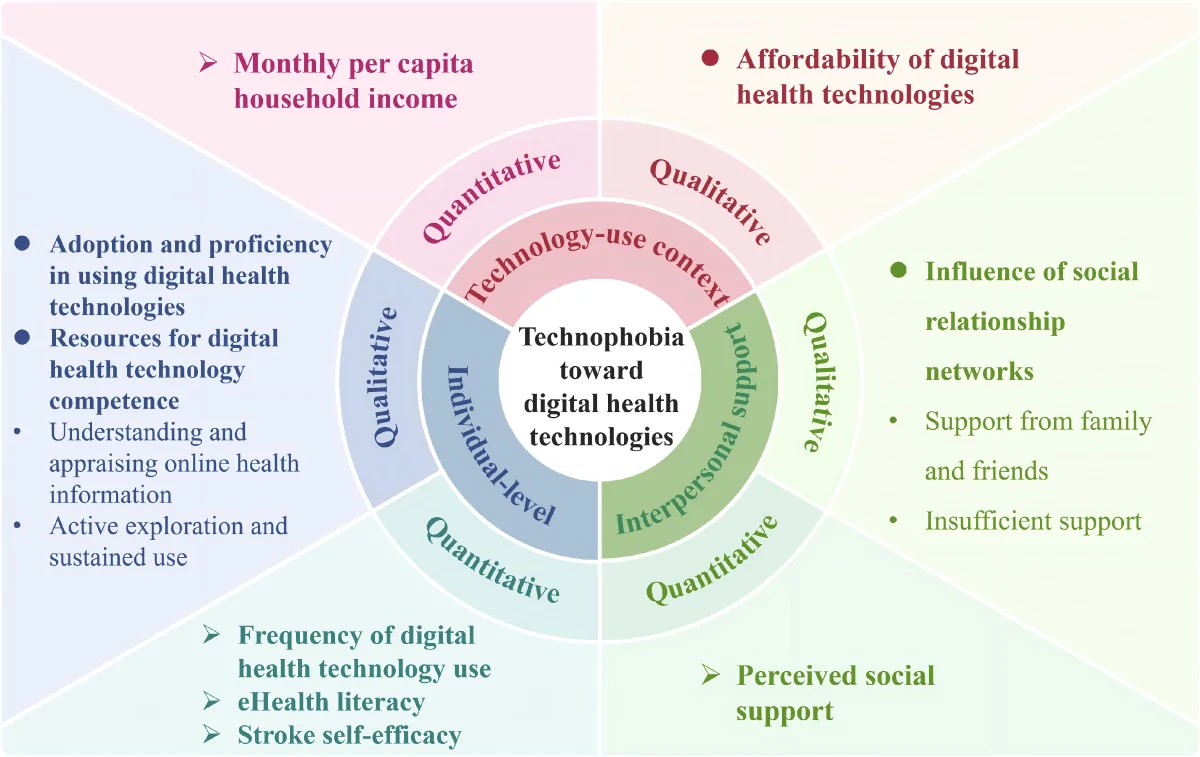
Socioecological map of the integrated quantitative and qualitative findings on technophobia toward digital health technologies among older patients with stroke.

Quantitative findings were integrated with the qualitative themes using the adapted socioecological perspective to examine convergence and complementarity across the individual, interpersonal support, and technology-use context domains. Figure 4 summarizes this integration; the detailed joint display is presented in Multimedia Appendix 5.

At the individual level, participants who used digital health technologies occasionally had lower technophobia scores than those who had never used such technologies. The qualitative findings provided convergent support for this categorical association, indicating that familiarity acquired through use was accompanied by greater understanding of and trust in digital health technologies. An additional finding that emerged inductively from the qualitative interviews was that some participants expressed willingness to explore AI-assisted health management. Higher eHealth literacy and stroke self-efficacy were similarly associated with lower technophobia scores. Qualitative analysis further indicated that difficulty appraising online health information was accompanied by concerns about information credibility, fraud, privacy, and security, whereas greater confidence in learning and problem solving was reflected in continued efforts to use digital health technologies and less apprehension during use.

At the interpersonal level, higher perceived social support was associated with lower technophobia scores. The qualitative findings further clarified this association, indicating that practical assistance, explanations, feedback, and reassurance from family members and others helped participants navigate operational uncertainty and alleviate fear during technology use. Interviews also revealed that, when such support was limited, some patients maintained a strong willingness to use digital health technologies but remained vulnerable to practical difficulties, often accompanied by emotional distress and frustration.

At the technology use context level, participants with a monthly per capita household income of Renminbi (Chinese yuan; RMB) 5000‐6999 (RMB 1=US $0.14 as of January 29, 2026) had lower technophobia scores than those in the reference category of less than RMB 1000. Qualitative findings concerning affordability helped contextualize this category-specific association: participants experiencing financial strain perceived operational errors as potential sources of additional expense and described heightened vigilance, hesitation, and tension when using digital health technologies. These accounts highlighted the perceived financial consequences of technology use as an important contextual dimension of technophobia toward digital health technologies.

Taken together, the integrated findings showed that the correlates and subjective experiences of technophobia toward digital health technologies spanned three socioecological domains: (1) individual capabilities and prior use experience, (2) interpersonal support, and (3) perceived financial consequences of technology use.

## Discussion

### Principal Findings and Comparison With Prior Work

The principal contribution of this study is its integration of quantitative and qualitative evidence to explain technophobia toward digital health technologies among older patients with stroke across three interrelated domains: (1) individual capabilities and prior experience, (2) interpersonal support, and (3) the context of technology use. The median technophobia score was 25.0 (IQR 22.0‐33.0), which was lower than the value reported by Peng et al [19] among community-dwelling older adults. This descriptive difference may be related to the greater willingness to use digital health technologies, greater prior exposure to such technologies, and more immediate access to support among participants in the hospital-based sample.

The findings further revealed an important tension within the concept of technophobia. Patients’ concerns about additional costs, inaccurate or misleading information, privacy breaches, and online fraud were grounded in real-world risks, while poststroke cognitive, executive, and communication impairments may increase their digital vulnerability [57,58]. Research involving people with acquired brain injury has likewise shown that cognitive and executive impairments are associated with susceptibility to online fraud and its financial and psychological consequences [59]. Technophobia may therefore comprise intertwined rational and irrational elements and may, in turn, increase patients’ risk of digital exclusion and their tendency to avoid digital health technologies [60]. Future research should deepen the theoretical interpretation of technophobia by building on its existing conceptualization as an irrational fear.

At the individual level, technophobia should not be equated with unwillingness to use digital health technologies. Although perceived usefulness was not directly assessed, the coexistence of technology-related concerns and willingness to use digital health technologies may be interpreted within the Technology Acceptance Model, which identifies perceived usefulness as an important contributor to behavioral intention [61]. eHealth literacy and stroke self-efficacy may represent conceptually distinct capability-related resources relevant to technophobia: the former concerns the ability to access, appraise, and apply digital health information, whereas the latter concerns confidence in performing poststroke activities and self-management tasks [62,63]. However, stroke self-efficacy is conceptually distinct from self-efficacy for using digital health technologies; their respective independent associations with technophobia require further investigation.

The unadjusted findings for age and self-rated health should be interpreted with caution. Although age is an important demographic correlate of technophobia, previous studies have reported inconsistent findings regarding the relationship between age and digital health literacy [64,65]. In this study, the weak association observed in the unadjusted analysis did not remain statistically significant after multivariable adjustment, suggesting that age may serve primarily as a marker of accumulated differences in experience with technology, capabilities, and support resources. Similarly, participants with better self-rated health had higher technophobia scores in the univariable analysis, but this difference was no longer statistically significant after adjustment. Previous research has also shown that the association between internet use and self-rated health may be influenced by socioeconomic and health-related factors [66,67], indicating a complex pattern of selection effects and confounding. In addition, patients with better self-rated health may have engaged with digital health technologies more independently and evaluated the associated risks themselves, whereas those in poorer health may have used these technologies less directly or relied more heavily on others.

At the interpersonal level, the observed association between perceived social support and technophobia was consistent with previous research [26]. This association may be understood through the stress-buffering hypothesis of social support [68]. When individuals encounter stressors related to digital health technologies, lower perceived social support may weaken the buffering effects of internal cognitive regulatory processes [69], intensify threat appraisal, and consequently exacerbate technophobia toward digital health technologies [70]. These findings suggest that technophobia toward digital health technologies among older patients with stroke should not be understood solely as a problem of person–technology fit but should also be considered within the context of the interpersonal support environment in which patients are embedded. The digital health equity framework further suggests that the availability of such support depends not only on patients’ family or social networks but also on whether health care systems incorporate accessible technology assistance into digital care pathways [71,72].

At the technology use context level, economic circumstances emerged as one of the factors associated with technophobia toward digital health technologies among older patients with stroke [21]. Ongoing medical and rehabilitation expenditures after stroke may increase the financial burden on patients with lower incomes [1], while the use and maintenance of digital health technologies themselves require financial investment and these technologies undergo rapid updates. Economic stratification within the older population may therefore contribute to intragenerational disparities in access, increasing the psychological burden associated with engaging with digital health technologies and thereby exacerbating technophobia toward these technologies [22].

### Implications for the Use of Digital Health Technologies in Stroke Rehabilitation

In the clinical implementation of digital health technologies, technophobia should not be used as an independent criterion for determining whether patients are suitable candidates for digital stroke care. Instead, it should be considered alongside eHealth literacy, prior technology use experience, stroke self-efficacy, and social support to identify the type and intensity of support required by individual patients. Assessment findings may inform the development of tiered support models, including independent use by patients, use with guidance from health care professionals, and shared use by patients and caregivers. Training should be centered on specific stroke rehabilitation or self-management tasks and incorporate practical demonstrations, repeated practice, and timely feedback. Digital health platforms should also adhere to principles of accessibility and usability, incorporating adaptive designs that accommodate poststroke impairments in language, cognition, vision, and motor function, while prioritizing information credibility, privacy protection, and safety as core design requirements [57].

Caregivers may be incorporated into the guidance and support processes for digital health technology use, provided that patients’ autonomy is respected and informed consent is obtained. Existing evidence suggests that telerehabilitation, remote training, and remote support are feasible approaches and may improve certain aspects of caregiver competence, self-efficacy, and health-related outcomes; however, findings regarding caregiver preparedness and caregiver burden remain inconsistent across studies, underscoring the continued need for technical assistance, digital skills training, and professional health care support [73,74]. Caregiver involvement may assist patients in understanding how to use digital health technologies, identifying trustworthy health information, and addressing problems encountered during use. For patients who lack family support or face a higher risk of digital exclusion, alternative pathways, including professional guidance, digital navigation support, in-person assistance, or other nondigital service options, should be considered to avoid making family support an implicit prerequisite for accessing digital health services [72].

The perspectives of older patients with stroke and their caregivers should be incorporated throughout the design, testing, and evaluation of digital health technologies. Patient advisory groups and participatory approaches can help identify needs that may not be captured through conventional usability assessments. Future research could integrate multimodal data, including behavioral logs, speech, images, and interaction trajectories, to characterize technology use under real-world conditions [75]. AI-assisted remote monitoring and virtual guidance may be prioritized for exploration among patients who have both the willingness and the requisite capacity to use these approaches, provided that professional oversight, algorithmic explainability, and clearly defined lines of responsibility are ensured [76].

At the health policy level, digital stroke programs should not be limited to providing patients with technology platforms but should incorporate caregiver training, community-based digital support, professional navigation services, and ongoing technical assistance into program design, workforce planning, and funding arrangements [73,77]. Governments, health care organizations, and payers should also consider the burdens imposed on patients by devices, internet connectivity, maintenance, and technical support, and explore sustainable reimbursement mechanisms that support digital follow-up, telerehabilitation, and digital navigation support. Procurement and regulatory criteria for digital health products could be further expanded to include stroke-specific accessibility, platform interoperability, privacy and data security, and AI governance requirements [78]. Beyond overall enrollment and usage rates, program evaluations should examine initial adoption, sustained engagement, and discontinuation patterns among patients with different levels of income, digital health literacy, functional status, and social support to determine whether digital services introduce new disparities in access.

### Limitations

This study has several limitations. First, recruitment from a single tertiary hospital in Shanghai may have introduced selection bias and limited the generalizability of the findings to community-dwelling stroke survivors and other care settings. Future multicenter studies should recruit participants from diverse hospital- and community-based settings across different geographic regions and health care contexts. Second, participation required individuals to understand the study content and to communicate their responses to the questionnaire or interview either in writing or orally. As a result, patients with severe cognitive, language, or communication impairments may have been underrepresented, and the findings may not have fully captured the features of technophobia among those unable to express their experiences with digital health technologies independently. Third, although overall poststroke disability and activities of daily living were assessed and analyzed using the mRS and Barthel Index, these measures may not adequately capture more specific functional dimensions closely related to digital health technology use. In particular, the severity of cognitive impairment among eligible participants, upper limb function, degree of care dependence, and access to digital devices were not systematically assessed as structured quantitative variables or incorporated into the statistical model. The absence of these variables may have resulted in residual confounding and limited the explanatory depth of the model with respect to technophobia and differences across patients with varying functional status. Future research should use multicenter, large-sample longitudinal designs and incorporate more specific stroke-related functional indicators and measures of digital device access to further validate and refine the present findings.

### Conclusions

This study showed that technophobia toward digital health technologies among older patients with stroke was inversely associated with monthly per capita household income, frequency of digital health technology use, perceived social support, eHealth literacy, and stroke self-efficacy. Qualitative findings further suggested that participants who used digital health technologies more frequently expressed greater acceptance of AI apps and a stronger willingness to use them. In addition, some participants who reported lower perceived social support still expressed a strong willingness to use digital health technologies. Future multicenter, large-sample longitudinal studies that include additional variables are needed to further validate and refine these findings.

## Supplementary material

10.2196/95921 Multimedia Appendix 1 GRAMMS, STROBE, and COREQ reporting checklists completed for this mixed methods study.

10.2196/95921 Multimedia Appendix 2 Coding scheme, model diagnostic plots, and complete multivariable linear regression results.

10.2196/95921 Multimedia Appendix 3 Semistructured interview guide used for qualitative data collection.

10.2196/95921 Multimedia Appendix 4 Characteristics of participants included in the qualitative phase (N=15).

10.2196/95921 Multimedia Appendix 5 Joint display integrating the main quantitative and qualitative findings across socioecological domains.

## Abbreviations

COREQ: Consolidated Criteria for Reporting Qualitative Research
eHEALS: eHealth Literacy Scale
GRAMMS: Good Reporting of A Mixed Methods Study
mHealth: mobile health
mRS: modified Rankin Scale
PSSS: Perceived Social Support Scale
RMB: Renminbi (Chinese yuan)
SSEQ: Stroke Self-Efficacy Questionnaire
STROBE: Strengthening the Reporting of Observational Studies in Epidemiology
TS: Technophobia Scale
