# Supplementary material for: Current Status and Associated Factors of Technophobia Toward Digital Health Technologies Among Older Patients With Stroke: Mixed Methods Study
Source: J Med Internet Res. 2026 Sep 25;28:e95921. doi: 10.2196/95921 (PMC13614213; doi:10.2196/95921)
Supplement: Supplementary file 1 [file jmir-v28-e95921-s001.docx]

# Multimedia Appendix 1. Checklists

**Table of Contents**

[Good reporting of a mixed-methods study (GRAMMS) checklist	2](#_Toc6987)

[STROBE Statement—Checklist of items that should be included in reports of](#_Toc20123) *[cross-sectional studies](#_Toc20123)*	[3](#_Toc20123)

[Standards for Reporting Qualitative Research (SRQR)	5](#_Toc8023)

## Good reporting of a mixed-methods study (GRAMMS) checklist

| **Guideline** | **Page information** |
| --- | --- |
| 1. Describe the justification for using a mixed methods approach to the research question | 4-5 |
| 2. Describe the design in terms of the purpose, priority and sequence of methods | 5-6 |
| 3. Describe each method in terms of sampling, data collection and analysis | 7-14 |
| 4. Describe where integration has occurred, how it has occurred and who has participated in it | 6, 14, 26-27, 35 |
| 5. Describe any limitation of one method associated with the present of the other method | 36 |
| 6. Describe any insights gained from mixing or integrating methods | 26-33 |

Reference: O'Cathain A, Murphy E, Nicholl J. The quality of mixed methods studies in health services research. J Health Serv Res Policy. 2008;13: 92-98.

## **STROBE** Statement—Checklist of items that should be included in reports of ***cross-sectional studies***

|  | Item No | **Recommendation** | **Page**  **No** |
| --- | --- | --- | --- |
| **Title and abstract** | 1 | (*a*) Indicate the study’s design with a commonly used term in the title or the abstract | 1 |
|  |  | (*b*) Provide in the abstract an informative and balanced summary of what was done and what was found | 1-2 |
| **Introduction** | | |  |
| Background/rationale | 2 | Explain the scientific background and rationale for the investigation being reported | 2-5 |
| Objectives | 3 | State specific objectives, including any prespecified hypotheses | 5 |
| **Methods** | | |  |
| Study design | 4 | Present key elements of study design early in the paper | 5 |
| Setting | 5 | Describe the setting, locations, and relevant dates, including periods of recruitment, exposure, follow-up, and data collection | 6 |
| Participants | 6 | (*a*) Give the eligibility criteria, and the sources and methods of selection of participants | 6 |
| Variables | 7 | Clearly define all outcomes, exposures, predictors, potential confounders, and effect modifiers. Give diagnostic criteria, if applicable | 7-8 |
| Data sources/ measurement | 8* | For each variable of interest, give sources of data and details of methods of assessment (measurement). Describe comparability of assessment methods if there is more than one group | 7-9 |
| Bias | 9 | Describe any efforts to address potential sources of bias | 6-9 |
| Study size | 10 | Explain how the study size was arrived at | 6 |
| Quantitative variables | 11 | Explain how quantitative variables were handled in the analyses. If applicable, describe which groupings were chosen and why | 7, 9-10, 16 |
| Statistical methods | 12 | (*a*) Describe all statistical methods, including those used to control for confounding | 9-10 |
|  |  | (*b*) Describe any methods used to examine subgroups and interactions | 9-10, 16-19 |
|  |  | (*c*) Explain how missing data were addressed | 9, 14 |
|  |  | (*d*) If applicable, describe analytical methods taking account of sampling strategy | N/A |
|  |  | (*e*) Describe any sensitivity analyses | N/A |
| **Results** | | |  |
| Participants | 13* | (a) Report numbers of individuals at each stage of study—eg numbers potentially eligible, examined for eligibility, confirmed eligible, included in the study, completing follow-up, and analysed | 14-15 |
|  |  | (b) Give reasons for non-participation at each stage | 14-15 |
|  |  | (c) Consider use of a flow diagram | 15 |
| Descriptive data | 14* | (a) Give characteristics of study participants (eg demographic, clinical, social) and information on exposures and potential confounders | 14-18 |
|  |  | (b) Indicate number of participants with missing data for each variable of interest | 14 |
| Outcome data | 15* | Report numbers of outcome events or summary measures | 17 |
| Main results | 16 | (*a*) Give unadjusted estimates and, if applicable, confounder-adjusted estimates and their precision (eg, 95% confidence interval). Make clear which confounders were adjusted for and why they were included | 10, 16, 18-21; Multimedia Appendix 2 |
|  |  | (*b*) Report category boundaries when continuous variables were categorized | 10, 16; Multimedia Appendix 2 |
|  |  | (*c*) If relevant, consider translating estimates of relative risk into absolute risk for a meaningful time period | N/A |
| Other analyses | 17 | Report other analyses done—eg analyses of subgroups and interactions, and sensitivity analyses | 16, 18-20 |
| **Discussion** | | |  |
| Key results | 18 | Summarise key results with reference to study objectives | 26-27, 32 |
| Limitations | 19 | Discuss limitations of the study, taking into account sources of potential bias or imprecision. Discuss both direction and magnitude of any potential bias | 31-32 |
| Interpretation | 20 | Give a cautious overall interpretation of results considering objectives, limitations, multiplicity of analyses, results from similar studies, and other relevant evidence | 27-31 |
| Generalisability | 21 | Discuss the generalisability (external validity) of the study results | 31 |
| **Other information** | | |  |
| Funding | 22 | Give the source of funding and the role of the funders for the present study and, if applicable, for the original study on which the present article is based | Title page; 32 |

*Give information separately for exposed and unexposed groups.

**Note:** An Explanation and Elaboration article discusses each checklist item and gives methodological background and published examples of transparent reporting. The STROBE checklist is best used in conjunction with this article (freely available on the Web sites of PLoS Medicine at http://www.plosmedicine.org/, Annals of Internal Medicine at http://www.annals.org/, and Epidemiology at http://www.epidem.com/). Information on the STROBE Initiative is available at www.strobe-statement.org.

## **Consolidated Criteria for Reporting Qualitative Research (COREQ) Checklist**

*A checklist of items that should be included in reports of qualitative research. You must report the page number in your manuscript where you consider each of the items listed in this checklist. If you have not included this information, either revise your manuscript accordingly before submitting or note N/A.*

**Domain 1: Research Team and Reflexivity**

| **No.** | **Item** | **Guide question / reporting element** | **Reported on page** |
| --- | --- | --- | --- |
| 1 | **Interviewer/facilitator** | Identify which author(s) conducted the interviews. | 11 |
| 2 | **Credentials** | State the interviewers’ academic or professional credentials. | 11 |
| 3 | **Occupation** | State the interviewers’ occupation or role when the study was conducted. | 11 |
| 4 | **Gender** | Report the interviewers’ gender. | 11 |
| 5 | **Experience and training** | Describe relevant qualitative research experience or training. | 11 |
| 6 | **Relationship established** | State whether a relationship with participants existed before recruitment. | 11 |
| 7 | **Participant knowledge of interviewer** | Describe what participants knew about the interviewers and study purpose. | 11 |
| 8 | **Interviewer characteristics** | Report relevant assumptions, interests, or characteristics that could shape the research. | 11-12 |

**Domain 2: Study Design**

| **No.** | **Item** | **Guide question / reporting element** | **Reported on page** |
| --- | --- | --- | --- |
| 9 | **Methodological orientation and theory** | Identify the methodological orientation underpinning the qualitative study. | 12 |
| 10 | **Sampling** | Describe how participants were selected. | 10-11 |
| 11 | **Method of approach** | Describe how potential participants were approached. | 11 |
| 12 | **Sample size** | Report the number of participants included. | 12, 21 |
| 13 | **Nonparticipation** | Report the number and reasons for refusal, nonparticipation, or dropout. | 15, Figure 2 |
| 14 | **Setting of data collection** | Describe where the interviews were conducted. | 11 |
| 15 | **Presence of nonparticipants** | State whether anyone other than the participant and interviewer was present. | 11 |
| 16 | **Description of sample** | Report characteristics relevant to interpreting the qualitative sample. | 21; Multimedia Appendix 4 |
| 17 | **Interview guide** | Describe the guide, prompts, development, review, and pilot testing. | 11-12; Multimedia Appendix 3 |
| 18 | **Repeat interviews** | State whether repeat interviews were conducted. | 11 |
| 19 | **Audio/visual recording** | State whether interviews were audio- or video-recorded. | 1 |
| 20 | **Field notes** | Describe field or reflexive notes made during or after interviews. | 11 |
| 21 | **Duration** | Report interview duration. | 12 |
| 22 | **Data saturation** | Describe how adequacy of the collected data and the stopping decision were assessed. | 12 |
| 23 | **Transcripts returned** | State whether transcripts were returned to participants for comment or correction. | 12 |

**Domain 3: Analysis and Findings**

| **No.** | **Item** | **Guide question / reporting element** | **Reported on page** |
| --- | --- | --- | --- |
| 24 | **Number of data coders** | Report how many researchers coded the data. | 12-13 |
| 25 | **Description of coding tree** | Describe the progression or structure linking codes, candidate themes, and final themes. | 12, 25 Multimedia Appendix 5 |
| 26 | **Derivation of themes** | State whether themes were prespecified or developed through analysis of the data. | 12 |
| 27 | **Software** | Identify software used to manage qualitative data. | 12 |
| 28 | **Participant checking** | State whether participants commented on preliminary findings or interpretations. | 12, 13 |
| 29 | **Quotations presented** | Provide participant quotations and identify each quotation consistently. | 22-24 |
| 30 | **Data and findings consistent** | Demonstrate consistency between presented evidence and analytic findings. | 21-24 |
| 31 | **Clarity of major themes** | Present the principal themes clearly. | 21-24 |
| 32 | **Clarity of minor themes** | Describe subthemes, diverse cases, or accounts that differ from dominant patterns. | 21–26 |

Reference: Tong A, Sainsbury P, Craig J. Consolidated criteria for reporting qualitative research (COREQ): a 32-item checklist for interviews and focus groups. Int J Qual Health Care. 2007;19(6):349-357. doi:10.1093/intqhc/mzm042.
