# Supplementary material for: Current Status and Associated Factors of Technophobia Toward Digital Health Technologies Among Older Patients With Stroke: Mixed Methods Study
Source: J Med Internet Res. 2026 Sep 25;28:e95921. doi: 10.2196/95921 (PMC13614213; doi:10.2196/95921)
Supplement: Supplementary file 2 [file jmir-v28-e95921-s002.docx]

# Multimedia Appendix 2. Supplementary Statistical Information for the Multivariable Linear Regression Analysis.

**Table of Contents**

[Table S1. Coding Scheme for Independent Variables.	2](#_Toc21254)

[Model Diagnostics	4](#_Toc5729)

[Table S2. Full results of the multivariable linear regression model examining factors associated with technophobia toward digital health technologies among older patients with stroke (N=343)](#_Toc15965)^[a](#_Toc15965)^[.	5](#_Toc15965)

## Table S1. Coding Scheme for Independent Variables.

| Variables | | Coding method |
| --- | --- | --- |
| **Education** | | |
|  | High school or below | 0 |
|  | Higher education or above | 1 |
| **Employment status** (dummy variables: Z1=On sick leave or retired; Z2=Unemployed) | | |
|  | Employed (reference) | (0, 0) |
|  | On sick leave or retired | (1, 0) |
|  | Unemployed | (0, 1) |
| **Monthly per capita household income (RMB^a^**, dummy variables: Z1=1000–2999; Z2=3000–4999; Z3=5000–6999; Z4=7000–9999; Z5=≥10000) | | |
|  | <1000^b^ (reference) | (0, 0, 0, 0, 0) |
|  | 1000-2999 | (1, 0, 0, 0, 0) |
|  | 3000-4999 | (0, 1, 0, 0, 0) |
|  | 5000-6999 | (0, 0, 1, 0, 0) |
|  | 7000-9999 | (0, 0, 0, 1, 0) |
|  | ≥10000 | (0, 0, 0, 0, 1) |
| **Type of medical coverage** (dummy variables: Z1=Urban employee medical insurance; Z2=New rural cooperative medical scheme) | | |
|  | Self-pay (reference) | (0, 0) |
|  | Urban employee medical insurance | (1, 0) |
|  | New rural cooperative medical scheme | (0, 1) |
| **Number of stroke episodes** (dummy variables: Z1=2, Z2=≥3) | | |
|  | 1 (reference) | (0, 0) |
|  | 2 | (1, 0) |
|  | ≥3 | (0, 1) |
| **Current health status** (dummy variables:Z1=Fair, Z2=Good) | | |
|  | Poor (reference) | (0, 0) |
|  | Fair | (1, 0) |
|  | Good | (0, 1) |
| **mRS^c^ score** (dummy variables: Z1=2–3, Z2=≥4) | | |
|  | 0–1 (reference) | (0, 0) |
|  | 2–3 | (1, 0) |
|  | ≥4 | (0, 1) |
| **Intention to use digital health technologies** | | |
|  | No | 0 |
|  | Yes | 1 |
| **Frequency of digital health technology use** (dummy variables: Z1=Occasionally, Z2=Sometimes, Z3=Often) | | |
|  | Never | (0, 0, 0) |
|  | Occasionally | (1, 0, 0) |
|  | Sometimes | (0, 1, 0) |
|  | Often | (0, 0, 1) |

**Table S1.** (continued)

| Variables | | Coding method |
| --- | --- | --- |
| **Use of health-related online media** | | |
|  | No | 0 |
|  | Yes | 1 |
| **Use of home health management devices** | | |
|  | No | 0 |
|  | Yes | 1 |
| Age (years) | | Continuous variable |
| eHealth literacy (score) | | Continuous variable |
| Perceived social support (score) | | Continuous variable |
| Stroke self-efficacy (score) | | Continuous variable |
| ^a^RMB: Renminbi (Chinese yuan).  ^b^RMB 1=US $0.14 as of January 29, 2026.  ^c^mRS: modified Rankin Scale. | | |

## Model Diagnostics

**Figure S1. Residual diagnostic plots for the multivariable linear regression model examining factors associated with technophobia toward digital health technologies (N=343). (A) Histogram of standardized residuals with a superimposed normal curve; (B) normal P–P plot of standardized residuals; and (C) scatterplot of standardized residuals against standardized predicted values.**

## Table S2. Full results of the multivariable linear regression model examining factors associated with technophobia toward digital health technologies among older patients with stroke (N=343)^a^.

| Variable | B^b^ | SE^b^ | Standardized β^b^ | t | 95% CI^b^ | P value | Tolerance | VIF^b^ |
| --- | --- | --- | --- | --- | --- | --- | --- | --- |
| (Intercept) | 75.01 | 5.04 | —^c^ | 14.89 | 65.09 to 84.92 | <.001 | — | — |
| **Education** |  |  |  |  |  |  |  |  |
| High school or below (reference) | — | — | — | — | — | — | — | — |
| Higher education or above | 0.05 | 0.79 | 0.00 | 0.07 | −1.51 to 1.61 | .948 | 0.805 | 1.243 |
| **Employment status** |  |  |  |  |  |  |  |  |
| Employed (reference) | — | — | — | — | — | — | — | — |
| On sick leave or retired | −2.44 | 1.58 | −0.08 | −1.55 | −5.54 to 0.66 | .122 | 0.394 | 2.540 |
| Unemployed | −0.31 | 2.03 | −0.01 | −0.15 | −4.30 to 3.69 | .879 | 0.444 | 2.253 |
| **Monthly per capita household income, RMB^d^** |  |  |  |  |  |  |  |  |
| <1000^e^ (reference) | — | — | — | — | — | — | — | — |
| 1000–2999 | −1.84 | 1.52 | −0.07 | −1.20 | −4.83 to 1.16 | .229 | 0.309 | 3.232 |
| 3000–4999 | −2.64 | 1.44 | −0.12 | −1.84 | −5.47 to 0.18 | .066 | 0.226 | 4.427 |
| 5000–6999 | −3.60 | 1.44 | −0.16 | −2.49 | −6.44 to −0.76 | .013 | 0.241 | 4.144 |
| 7000–9999 | −2.27 | 1.80 | −0.06 | −1.26 | −5.81 to 1.27 | .208 | 0.463 | 2.159 |
| ≥10,000 | −3.07 | 2.20 | −0.06 | −1.39 | −7.39 to 1.26 | .164 | 0.596 | 1.677 |
| **Type of medical coverage** |  |  |  |  |  |  |  |  |
| Self-pay (reference) | — | — | — | — | — | — | — | — |
| Urban employee medical insurance | 1.63 | 1.57 | 0.06 | 1.04 | −1.45 to 4.71 | .300 | 0.256 | 3.909 |
| New Rural Cooperative Medical Scheme | −3.08 | 1.73 | −0.11 | −1.78 | −6.49 to 0.33 | .077 | 0.256 | 3.909 |

| **Table S2.** (continued) | | | | | | | | |
| --- | --- | --- | --- | --- | --- | --- | --- | --- |
| Variable | B^b^ | SE^b^ | Standardized β^b^ | t | 95% CI^b^ | P value | Tolerance | VIF^b^ |
| **Number of stroke episodes** |  |  |  |  |  |  |  |  |
| 1 (reference) | — | — | — | — | — | — | — | — |
| 2 | 0.08 | 0.86 | 0.00 | 0.09 | −1.61 to 1.77 | .925 | 0.906 | 1.103 |
| ≥3 | −0.89 | 1.24 | −0.02 | −0.72 | −3.33 to 1.55 | .473 | 0.855 | 1.170 |
| **Current health status** |  |  |  |  |  |  |  |  |
| Poor (reference) | — | — | — | — | — | — | — | — |
| Fair | −0.17 | 0.92 | −0.01 | −0.18 | −1.97 to 1.63 | .854 | 0.604 | 1.656 |
| Good | 0.25 | 1.07 | 0.01 | 0.23 | −1.86 to 2.36 | .815 | 0.617 | 1.622 |
| **mRS^f^ score** |  |  |  |  |  |  |  |  |
| 0–1 (reference) | — | — | — | — | — | — | — | — |
| 2–3 | −0.16 | 0.95 | −0.01 | −0.17 | −2.03 to 1.71 | .866 | 0.471 | 2.123 |
| ≥4 | −1.74 | 1.28 | −0.07 | −1.35 | −4.26 to 0.79 | .177 | 0.421 | 2.374 |
| **Intention to use digital health technologies** |  |  |  |  |  |  |  |  |
| No (reference) | — | — | — | — | — | — | — | — |
| Yes | 0.83 | 1.26 | 0.03 | 0.65 | −1.66 to 3.31 | .513 | 0.415 | 2.410 |
| **Frequency of digital health technology use** |  |  |  |  |  |  |  |  |
| Never (reference) | — | — | — | — | — | — | — | — |
| Occasionally | −3.98 | 1.50 | −0.19 | −2.65 | −6.94 to −1.03 | .008 | 0.200 | 4.992 |
| Sometimes | −2.58 | 1.65 | −0.12 | −1.56 | −5.82 to 0.67 | .119 | 0.159 | 6.301 |
| Often | −2.70 | 1.89 | −0.08 | −1.43 | −6.42 to 1.02 | .155 | 0.305 | 3.273 |
| **Use of health-related online media** |  |  |  |  |  |  |  |  |
| No (reference) | — | — | — | — | — | — | — | — |
| Yes | −0.20 | 1.11 | −0.01 | −0.18 | −2.37 to 1.98 | .860 | 0.343 | 2.917 |

| **Table S2.** (continued) |  |  |  |  |  |  |  |  |
| --- | --- | --- | --- | --- | --- | --- | --- | --- |
| Variable | B^b^ | SE^b^ | Standardized β^b^ | t | 95% CI^b^ | P value | Tolerance | VIF^b^ |
| **Use of home health management devices** |  |  |  |  |  |  |  |  |
| No (reference) | — | — | — | — | — | — | — | — |
| Yes | −1.64 | 1.15 | −0.08 | −1.43 | −3.91 to 0.62 | .155 | 0.324 | 3.083 |
| Age, years | 0.01 | 0.05 | 0.01 | 0.26 | −0.09 to 0.12 | .795 | 0.792 | 1.262 |
| eHealth literacy score | −0.45 | 0.07 | −0.38 | −6.82 | −0.58 to −0.32 | <.001 | 0.323 | 3.096 |
| Perceived social support score | −0.35 | 0.03 | −0.49 | −10.63 | −0.42 to −0.29 | <.001 | 0.469 | 2.132 |
| Stroke self-efficacy score | −0.08 | 0.03 | −0.13 | −2.60 | −0.13 to −0.02 | .010 | 0.404 | 2.478 |

^a^*R*=0.83, *R²*=0.69, Adjusted *R²*=0.66, *F*=26.87, *P*<.001.

^b^B: unstandardized regression coefficient; SE: standard error; β: standardized regression coefficient; CI: confidence interval; VIF: variance inflation factor.

^c^Not applicable.

^d^RMB: Renminbi (Chinese yuan).

^e^RMB 1=US $0.14 as of January 29, 2026.

^f^mRS: modified Rankin Scale.
