# Supplementary material for: Current Status and Associated Factors of Technophobia Toward Digital Health Technologies Among Older Patients With Stroke: Mixed Methods Study
Source: J Med Internet Res. 2026 Sep 25;28:e95921. doi: 10.2196/95921 (PMC13614213; doi:10.2196/95921)
Supplement: Supplementary file 3 [file jmir-v28-e95921-s003.docx]

# Multimedia Appendix 3. Topic-guide for qualitative interviews.

A. **General questions**

- Do you currently use any digital health technologies or services (e.g., online appointment booking, online follow-up consultations, or health management applications)?
- In your opinion, what are the main ways in which digital health technologies support healthcare access, rehabilitation, or health self-management?

B. **In-depth questions**

- While using digital health technologies, have you ever felt tense, worried, or uneasy? Please describe an experience that you recall clearly, including the specific context and what you think contributed to these feelings.
- What difficulties or concerns have you encountered during use? How have these factors influenced your use (e.g., reduced use, avoidance, or reliance on others)
- When obtaining health information online or using online health services, do you experience difficulties in understanding, judging, or selecting information/services? How do you usually determine whether the information or service is reliable?
- How confident do you feel about completing relevant tasks independently? When problems arise, how do you typically deal with them?
- When you find a technology difficult to use or feel reluctant to use it, who do you usually ask for help? What kind of assistance do they provide? Does this support affect how tense or worried you feel?
- Do factors such as access to devices, internet connectivity, or costs affect your use or increase your concerns?
- Have any post-stroke physical or cognitive changes affected your use of digital health technologies? If so, in what ways?

C. **Concluding question**

- To reduce feelings of tension or worry and to increase willingness to use digital health technologies, what improvements would you like to see in future digital health technologies or services
