# Supplementary material for: Current Status and Associated Factors of Technophobia Toward Digital Health Technologies Among Older Patients With Stroke: Mixed Methods Study
Source: J Med Internet Res. 2026 Sep 25;28:e95921. doi: 10.2196/95921 (PMC13614213; doi:10.2196/95921)
Supplement: Supplementary file 4 [file jmir-v28-e95921-s004.docx]

# Multimedia Appendix 4. Participant characteristics in the qualitative phase (N=15).

| Number | Age (years) | Gender | Disease duration (months) | Education | Stroke type | mRS^a^ score | Monthly per capita household income (RMB^b^) | Frequency of digital health technology use | Technophobia score |
| --- | --- | --- | --- | --- | --- | --- | --- | --- | --- |
| N1 | 65 | Male | 22 | High school or below | Ischemic stroke | 3 | 1000–2999^c^ | Never | 35 |
| N2 | 70 | Female | 11 | High school or below | Hemorrhagic stroke | 3 | 1000–2999 | Occasionally | 33 |
| N3 | 65 | Female | 36 | Higher education or above | Hemorrhagic stroke | 2 | 5000–6999 | Sometimes | 31 |
| N4 | 61 | Male | 25 | Higher education or above | Ischemic stroke | 1 | ≥10000 | Often | 17 |
| N5 | 69 | Male | 16 | High school or below | Ischemic stroke | 2 | 3000–4999 | Sometimes | 25 |
| N6 | 72 | Female | 48 | Higher education or above | Ischemic stroke | 1 | 7000–9999 | Sometimes | 22 |
| N7 | 66 | Male | 17 | High school or below | Hemorrhagic stroke | 3 | 3000–4999 | Occasionally | 32 |
| N8 | 80 | Female | 38 | High school or below | Ischemic stroke | 3 | 5000–6999 | Occasionally | 29 |
| N9 | 67 | Female | 6 | High school or below | Ischemic stroke | 2 | 1000–2999 | Occasionally | 30 |
| N10 | 76 | Male | 24 | High school or below | Hemorrhagic stroke | 4 | 3000–4999 | Never | 48 |
| N11 | 64 | Male | 12 | High school or below | Ischemic stroke | 4 | <1000 | Never | 50 |
| N12 | 78 | Male | 26 | High school or below | Ischemic stroke | 3 | 3000–4999 | Occasionally | 28 |
| N13 | 71 | Male | 33 | Higher education or above | Mixed stroke | 2 | 5000–6999 | Often | 24 |
| N14 | 74 | Female | 20 | Higher education or above | Ischemic stroke | 1 | 3000–4999 | Sometimes | 21 |
| N15 | 68 | Female | 15 | Higher education or above | Ischemic stroke | 1 | 3000–4999 | Often | 18 |

^a^mRS: modified Rankin Scale.

^b^RMB: Renminbi (Chinese yuan).

^c^RMB 1=US $0.14 as of January 29, 2026.
