# Supplementary material for: Current Status and Associated Factors of Technophobia Toward Digital Health Technologies Among Older Patients With Stroke: Mixed Methods Study
Source: J Med Internet Res. 2026 Sep 25;28:e95921. doi: 10.2196/95921 (PMC13614213; doi:10.2196/95921)
Supplement: Supplementary file 5 [file jmir-v28-e95921-s005.docx]

**Multimedia Appendix 5.** Joint display of the main quantitative and qualitative findings.

| Socioecological domain | Main quantitative findings | Main qualitative findings | | | Integrated findings |
| --- | --- | --- | --- | --- | --- |
|  |  | Categories | | Representative quotes |  |
| **Technology-**  **use context** | **Monthly per capita household income (RMB^a^)**   - 5000–6999^b^:   (*B*=−3.60, 95% CI −6.44 to −0.76, *P*=.013) | **Category 1. Affordability of Digital Health Technologies** | | | - **Confirmation:**   Lower monthly per-capita household income was associated with higher levels of technophobia toward digital health technologies. |
|  |  |  | - “…Since I got sick, every follow-up costs money, such as tests, appointments, and all of it. Treatment and rehab have already put me under a lot of financial pressure, so I have to stretch every penny. That’s why whenever I use anything medical online, I get really anxious and extra careful. I’m afraid I’ll hit the wrong thing by accident and end up getting charged for no reason…” (Male, aged 65 years) - “Rehab bills have been weighing on me, and I have to watch every penny. So whenever I use my phone to register or make a payment, I get really tense. I move really slowly and check it over and over before I dare hit ‘confirm’, I’m scared I’ll tap the wrong button and the money will be taken for nothing...” (Female, aged 68 years) | |  |
| **Individual** | **Frequency of digital health technology use**   - Occasionally^c^:   (*B*=−3.98, 95% CI −6.94 to −1.03, *P*=.008) | **Category 2. Adoption and Proficiency in Using Digital Health Technologies** | | | - **Confirmation:**   Higher frequency of digital health technology use was associated with lower levels of technophobia toward digital health technologies.   - **Expansion:**   Among participants with higher frequency of digital health technology use, some reported greater acceptance of AI applications and a more proactive intention to use them. |
|  |  |  | - “…I use WeChat official accounts a lot to look up stuff about stroke. Some of them are really good. I’ve also learned how to book an appointment through the hospital’s official account and check my test results. I can even see the charges from when I was in the hospital… The more you use it, the more you get the hang of it… And lately I’ve started trying AI to answer some of my questions. That AI search feels more thorough than when I look things up myself, and the suggestions are a bit more to the point.” (Male, aged 61 years) | |  |

**Multimedia Appendix 5.** Continued

| Socioecological domain | Main quantitative findings | Main qualitative findings | | Integrated findings |
| --- | --- | --- | --- | --- |
|  |  | Categories | Representative quotes |  |
| **Interpersonal support** | **Perceived social support**^d^  (*B*=−0.35, 95% CI −0.42 to −0.29, *P*<.001) | **Category 3. Influence of Social Relationship Networks** | | - **Confirmation:**   Higher levels of perceived social support were associated with lower levels of technophobia toward digital health technologies.   - **Expansion:**   Despite low perceived social support, some patients still reported relatively high intentions to use digital health technologies. |
|  |  | - *Support From Family and Friends* | - “Just having someone who really gets it standing next to you makes you feel a lot more at ease. Even if you hit the wrong button or the page gets stuck, they can check it right away and tell you what to do next. And then, using these new things doesn’t feel nearly as scary…” (Female, aged 65 years) - “Now I’ve added quite a few other stroke patients on WeChat. Most days, we just chat in the group about how we’re doing and how our recovery’s been lately. If someone has something new going on or has found a good tip, they’ll share it with everyone. When there are features I don’t really understand, I’ll turn around and ask my kids. They’re young, so they know this stuff, and little by little I’ve picked up some things too. Back and forth like that, it feels like I’m in touch with people way more than before…” (Male, aged 71 years) |  |
|  |  | - *Insufficient Support* | - “I want to use it, and I want to learn, but there’s no one to teach me. My kids are all busy, and whenever I’m in the hospital, I’m tied up with rehab, so I don’t really have time to ask the doctors or nurses. I don’t have time to learn from other patients either, or just talk things through. No one shows me, and some of it I honestly can’t make sense of at all—I can’t learn it. And it really stresses me out…” (Male, aged 78 years) |  |

**Multimedia Appendix 5.** Continued

| Socioecological domain | Main quantitative findings | Main qualitative findings | | Integrated findings |
| --- | --- | --- | --- | --- |
|  |  | Categories | Representative quotes |  |
| **Individual** | **eHealth literacy**^d^  (*B*=−0.45, 95% CI −0.58 to −0.32, *P*<.001) | **Category 4. Resources for Digital Health Technology Competence** | | - **Confirmation:**   Lower eHealth literacy was associated with higher levels of technophobia toward digital health technologies. |
|  |  | - *Understanding and Appraising Online Health Information* | - “Once my arm on the weak side started feeling numb, I went online to see what it might be. I wasn’t really paying attention and clicked a pop-up by mistake, and that afternoon someone called me asking if I wanted to buy some ‘special stroke medicine.’ It felt like the moment I went online, my number got out. Since then, I haven’t dared to just use those online consultations anymore.” (Female, aged 80 years) |  |
| **Individual** | **Stroke self-efficacy**^d^  (*B*=−0.08, 95% CI −0.13 to −0.02, *P*=.010) | **Category 4. Resources for Digital Health Technology Competence** | | - **Confirmation:**   Higher self-efficacy was associated with lower levels of technophobia toward digital health technologies. |
|  |  | - *Active Exploration and Sustained Use* | - “Since I got sick, I’ve been using my phone to learn about stroke. I’ve taken quite a few health education classes and read a lot of information. Through this, I’ve got to know my own condition better and learned how to use these digital tools to help with my daily rehab. Now, when I use them, I feel much more at ease; they don’t seem that complicated anymore. If I take it step by step, I feel I can learn how to use them.” (Female, aged 72 years) |  |

^a^RMB: Renminbi (Chinese yuan); RMB 1=US $0.14 as of January 29, 2026.

^b^Reference: <1000

^c^Reference: Never

^d^Continuous variable
